# Development and evaluation of an artificial intelligence for bacterial growth monitoring in clinical bacteriology

**DOI:** 10.1128/jcm.01651-23

**Published:** 2024-04-04

**Authors:** Damien Jacot, Shklqim Gizha, Cedrick Orny, Mathieu Fernandes, Carmelo Tricoli, Raphael Marcelpoil, Guy Prod'hom, Jean-Marc Volle, Gilbert Greub, Antony Croxatto

**Affiliations:** 1 Institute of Microbiology, Lausanne University Hospital and University of Lausanne, Lausanne, Switzerland; 2 Becton Dickinson Kiestra, Le Pont-de-Claix, France; 3 Infectious Diseases Service, Department of Medicine, Lausanne University Hospital and University of Lausanne, Lausanne, Switzerland; 4 ADMED, Department of Microbiology, La Chaux-de-Fonds, Switzerland; Medical College of Wisconsin, Milwaukee, Wisconsin, USA

**Keywords:** bacteriology, artificial intelligence, growth monitoring, sterile plates

## Abstract

In clinical bacteriology laboratories, reading and processing of sterile plates remain a significant part of the routine workload (30%–40% of the plates). Here, an algorithm was developed for bacterial growth detection starting with any type of specimens and using the most common media in bacteriology. The growth prediction performance of the algorithm for automatic processing of sterile plates was evaluated not only at 18–24 h and 48 h but also at earlier timepoints toward the development of an early growth monitoring system. A total of 3,844 plates inoculated with representative clinical specimens were used. The plates were imaged 15 times, and two different microbiologists read the images randomly and independently, creating 99,944 human ground truths. The algorithm was able, at 48 h, to discriminate growth from no growth with a sensitivity of 99.80% (five false-negative [FN] plates out of 3,844) and a specificity of 91.97%. At 24 h, sensitivity and specificity reached 99.08% and 93.37%, respectively. Interestingly, during human truth reading, growth was reported as early as 4 h, while at 6 h, half of the positive plates were already showing some growth. In this context, automated early growth monitoring in case of normally sterile samples is envisioned to provide added value to the microbiologists, enabling them to prioritize reading and to communicate early detection of bacterial growth to the clinicians.

## INTRODUCTION

Clinical bacteriology laboratories have experienced a revolution with the introduction of fully automated systems (1–6). Inoculation, plate transport, and image digitalization were among the first innovations with the reading now performed on digital images where microbiologists select microbial colonies for subsequent follow-up actions (7, 8). Development of intelligent algorithms for plates reading linked to expert systems will further provide a fully automated approach to the bacteriology workflow. Although presumptive identification of microbial colonies on complex specimens remains a future but ongoing prospect (9), other simpler tasks were and would be automatized. Currently, the imaging applications available are mostly restricted to urine specimens as either stand-alone instrument or coupled to automated systems, such as the BD Kiestra TLA from Becton, Dickinson and Company (BD) and the WASPLab from Copan (10–16). These automated systems were developed on urine cultures only and to allow a semi-quantification and the identification of sterile plates on a set of dedicated media. In addition, some algorithms were developed toward specific screens as, for example, to selectively detect group B *Streptococcus* (17, 18), vancomycin-resistant *Enterococcus* (VRE) (19, 20), methicillin-resistant *Staphylococcus aureus* (MRSA) (21, 22), or *Streptococcus pyogenes* pharyngitis (23).

Depending on the laboratory setting, sterile plates could account for a significant amount of the workload. Therefore, imaging applications dedicated to the identification of growth and the release of sterile plates on any type of specimens and media have the potential to greatly improve laboratory productivity. The growth detection performance of the algorithm was therefore investigated on different specimens and on five different media. The developed artificial intelligence (AI) was not only investigated at the usual reading timepoints in microbiology (18–24 h and/or 48 h) but every 2 h starting from the inoculation step. This study evaluated the algorithm performance with the intent of (i) releasing sterile plates at pre-determined timepoints and (ii) monitoring early growth on specific specimen types.

## MATERIALS AND METHODS

### Laboratory automation environment

All samples were processed on the BD Kiestra Total Lab Automation (TLA) System (BD Kiestra, Drachten, the Netherlands) with the following modules: BD Kiestra SorterA, BD Kiestra BarcodA, BD Kiestra InoqulA+, BD Kiestra ProceedA conveyor, BD Kiestra ReadA Compact incubators, and seven BD Kiestra ErgonomicA working stations.

### Specimen selection

A representative selection of clinical specimens (Table 1) from outpatients and hospitalized patients at the University Hospital of Lausanne (CHUV, Switzerland) was used over a 1-yr period (December 2019 to February 2021). Quotas of specimen types were defined at the beginning of the study without exclusion criteria. All specimens were irreversibly anonymized before entering the study. All subcultures originated from anonymized clinical specimens. For blood cultures, subcultures were performed directly from patient’s positive bottles and immediately anonymized. As per this study design, no ethical request was required.

**TABLE 1:** Description of the specimens and plates used for validation^*a*^

Specimen/plate	CHOC	ORI	CNA	COL	MAC	Sum
Blood culture	86	84	80	85	86	421
Respiratory	168	169	165	167	171	840
Tissue and pus	190	197	188	189	193	957
Superficial wound	32	28	31	31	28	150
ENT	21	17	20	19	19	96
Urine	193	191	196	195	195	970
Urogenital	82	80	84	83	81	410
Sum	772	766	764	769	773	3,844

^*a*^ No growth was found by the human on 36% of the plates investigated. ENT: ear, nose, and throat.

### Plated media

BD CHROMagar Orientation (ORI), Columbia Agar with 5% Sheep Blood Plate (COL), BD Chocolate II Agar (CHOC), BD MacConkey Agar (MAC), and BD Columbia CNA Agar (CNA) media were used. The BD BBL Chocolate II Agar with Bacitracin (CHOC-B, abbreviated in the paper as CHOC) was used instead of the BD BBL Chocolate II Agar for all respiratory specimens.

### Inoculation and incubation

Samples were inoculated with the BD Kiestra InoqulA+. Urine specimens were streaked using the 04 Zigzag 2.5-1 INOC5TREAK 5200 pattern, while all other specimens were streaked with the 20 Multizone 3 repeats. After automated inoculation, plates were immediately incubated at 37°C in normal or in 5% CO_2_ atmosphere incubators (BD Kiestra ReadA Compact).

### Imaging acquisition

The image acquisition was performed with the BD Kiestra ReadA Compact using the OPTIS software (BD Kiestra, Drachten, the Netherlands). Images were captured at the following timepoints: 1, 2, 4, 6, 8, 10, 12, 14, 16, 18, 20, 22, 24, 36, and 48 h.

### Plate review

The images were reviewed using Kiestra *CIS*, a BD-developed software for petri dishes images annotation. Each timepoint was reviewed by two reviewers out of a pool of 25 microbiologists. In case of discrepancies (3.2%), a third reviewer was involved for arbitration. The images were randomly and independently presented to the reviewers, but the reviewers could access all timepoints. Possible answers were growth or no growth.

### Image analysis and software development

The growth monitoring AI algorithm (not available for clinical use) is an extension of BD Kiestra Urine Culture Application (UCA) and has been trained using seeded and urine samples, and 3, 12, and 24 h incubation images on BD BBL Columbia CNA Agar, BD BBL Columbia and Tryptic Soy Agar II, BD BBL CHROMagar Orientation, BD BBL MacConkey II Agar, BD BBL CLED Agar, and BD CHOC plates acquired prior to the study. Data acquired during this study have been solely used for AI performance investigation. For the algorithmic analysis, t = 1 h and t = 2 h were chosen as a reference for plate background calculation. Time series containing images failing AI image adequacy check were discarded (e.g., bead on the plate, insufficient image quality). The algorithm detects colony forming unit (CFU) candidates or artefacts in the image by looking for image areas that change over the incubation course.

### Plate analysis

Four thousand one hundred fifty-one plates were analyzed by the growth/no growth monitoring AI. Forty-nine plates with incomplete time series, 214 plates with more than 2-h delay from the expected time of image acquisition (this exclusion criterion was not used for the plates imaged at 36 and 48 h), and 41 plates with incoherent (multiple discordances between microbiologists) and three incomplete truths (review was missing) were removed. Three thousand eight hundred forty-four plates were kept for final analysis. Thirty-eight plates with a non-monotonic human truth behavior (i.e., a growth followed by a no growth statement) were manually curated by the consensus of two additional reviewers to obtain a monotonic truth.

### Statistical analyses

Data analyses were performed with R 4.0.2 using the following packages: epiR (2.0.57) for sensitivity, specificity, and negative and positive predictive value; ggplot2 (3.3.3), caret (6.0-92), and heatmap (1.0.12) for the plots, confusion, and time matrices, respectively. The function wilcox.test from the library stats (3.6.2) was used for Wilcoxon signed-rank tests.

## RESULTS

### Performance of the AI algorithm

**Fig 1:**
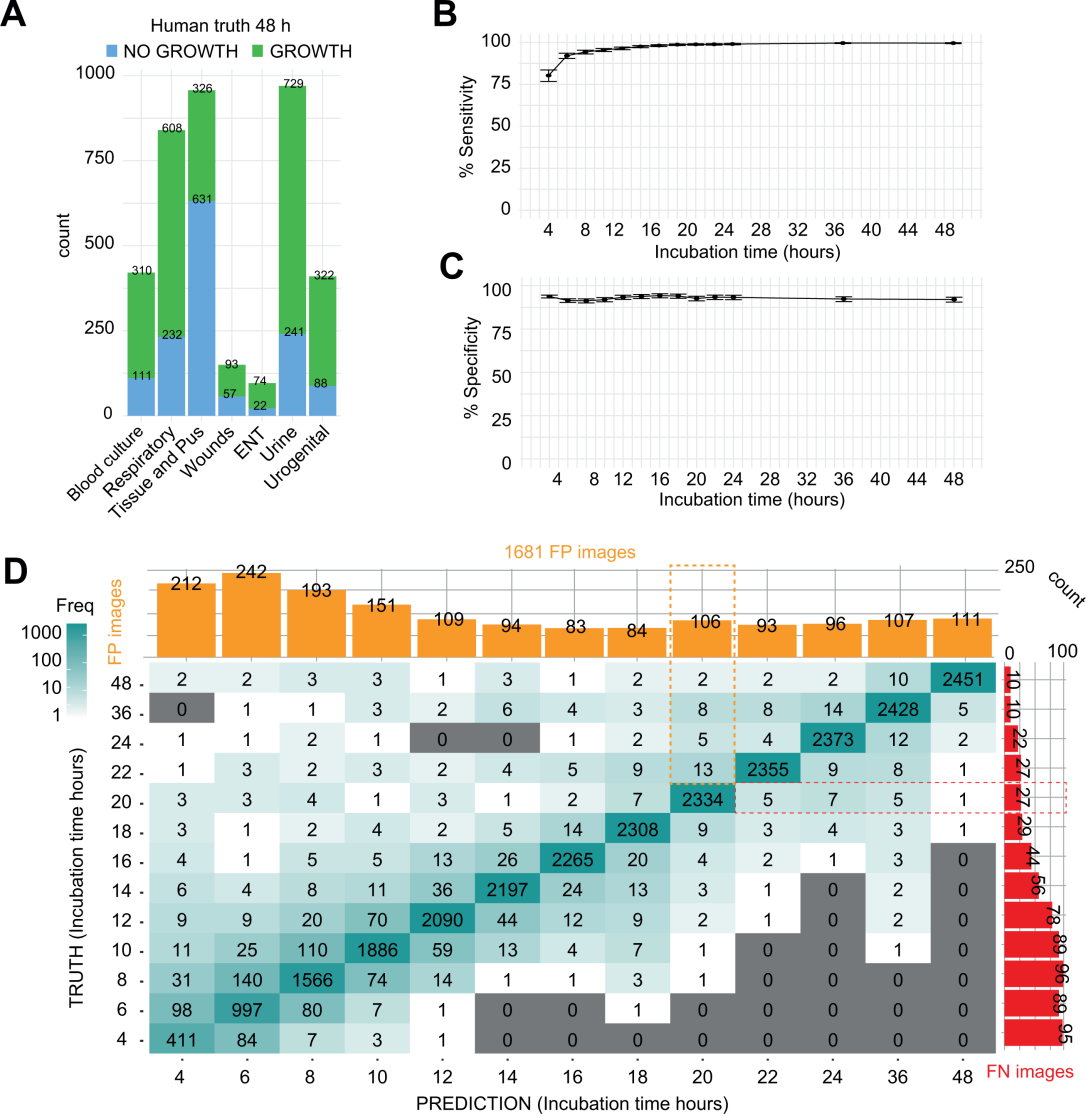
(A) Number of plates with no growth or growth (human truth at 48 h) among all specimens used in the study. (**B and C**) Overall, sensitivity increased over time while specificity remained constant. Error bars represent 95% CI. (**D**) Confusion matrix, compared at all timepoints, and the prediction of growth of the AI (*x* axis) against the human (*y* axis). The number of FP and FN prediction is respectively shown in orange and red plots. At 48 h, 2,451 plates showed a concordant growth prediction, while 5 were not predicted by the AI and 111 were FP calls. A reading at 20 h by the AI shows 2,334 perfect matches between the human and the AI, with 27 FN and 93 FP calls. Out of the 27 FN, 5 are later predicted at 22 h, 7 at 24 h, 5 at 36 h, and 1 at 48 h (red dashed lines). Conversely, out of the 93 FP calls, 13 were stated as growth by the human at 22 h, 5 at 24 h, 8 at 36 h, and 2 at 48 h (orange dashed lines). A detailed explanation on how the matrix is generated is provided in Figure S1E through J.

The growth or no growth monitoring AI algorithm was developed for (i) bacterial growth detection in the perspective of an automatic processing of sterile plates (i.e., identification and discharge by the automated systems BD Kiestra TLA of the culture if negative) and for (ii) early growth detection. Performances of the algorithm were evaluated on 3,844 plates (five media) inoculated with representative clinical specimens (Table 1; Fig. 1A). Plates were imaged 15 times (49,972 timepoints) and read by two different microbiologists, creating 99,944 human truths used as gold standard for algorithm prediction (growth or no growth).

Without additional data curation, a sensitivity of 97.44% (97.25%–97.63%), a specificity of 92.89% (92.56%–93.21%), and an accuracy of 94.5% (confidence interval [CI] 94.2%–94.8%) was observed when the overall performance with all timepoints was compared. As expected, sensitivity increased over time, reaching 99.59% (95% CI: 99.25%–99.81%) at 48 h (Fig. 1B; Table S1), while specificity remained relatively constant (91.97%, 95% CI: 90.41%–93.35% at 48 h) (Fig. 1C; Table S1). No significant difference was observed between the different agar plates except for the CHOC, which showed a lower specificity (Fig. S1A and B; Table S2). Similarly, specimen types showed comparable performances except for the urogenital specimens in which a lower specificity was observed (Fig. S1C and D; Table S3).

To further dissect the algorithmic predictions, a confusion matrix, in which every human truth was compared to its corresponding AI prediction, was generated (Fig. 1D; Fig. S1E through J). The diagonal represents perfect matches between the AI prediction and the human, while everything above or below represents respectively false-positive (FP) or false-negative (FN) AI predictions. At a given timepoint, an image could be predicted as an FN (below the diagonal) while being a true positive (TP) the next timepoint with therefore only a late algorithmic prediction. Conversely, the same holds true for an early FP prediction (above the diagonal). The FN (orange) and FP (red) plots represent the sum of all incorrect predictions of the AI at each timepoint.

The confusion matrix showed discrepancies at early timepoints corresponding to inconsistencies between the algorithm and the humans when the biomass is low and more difficult to discriminate. Accordingly, most of the FP calls were generated at an early timepoint (Fig. 1D, orange plot) and then remained constant with 111 FN plates generated at 48 h, while 2,451 plates were correctly predicted as growth. Importantly, the FN predictions decreased through time with only 10 FN plates at 48 h (Fig. 1D, red plot).

### False-negative predictions

**Fig 2:**
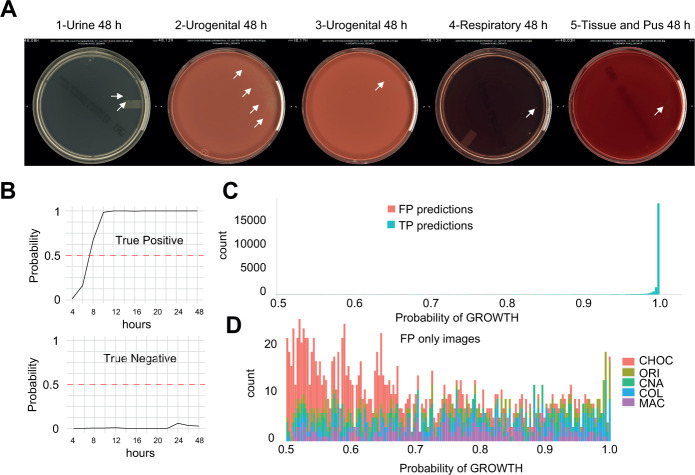
(A) Images showing the five FN plates at 48 h. Colonies misidentified by the AI are shown with white arrows. (B) Typical probability plot of TP and TN predictions by the AI. A probability higher than 0.5 is considered as growth. (C) Histogram showing the distribution of growth predictions by the AI (>0.5) for both the FP (*n* = 1,681) and TP (*n* = 25,655) calls. (D) Zoom on the FP predictions only where the CHOC plates accumulate with a low prediction of growth.

Re-examination of the 10 FN plates (Fig. S2) by two microbiologists showed that five were actual FN (Table S4). Indeed, the truth detected in the other plates was artefacts already present at the initial timepoints and misidentified by the human during the initial review. Therefore, the corrected sensitivity at 48 h reached 99.80% (99.53%–99.99%). Out of the 5 remaining FN plates, 3 contained one single colony, 1 two colonies, and 1 showed a faint monolayer of possible *Lactobacillus* species with poor contrast on the CHOC plate (Fig. 2A). Although clinically irrelevant here (urogenital specimen), *Lactobacillus* are known to be implicated, for example, in liver abscess. For the tissue and pus specimen in which any type of growth would be significant, a single small alpha-hemolytic colony next to a plate artefact was missed by the AI.

In routine clinical microbiology, plates are commonly examined twice, once at 18–24 h and again at 36–48 h. However, depending on the clinical specimen, only one reading at 18–24 h could be performed. At the CHUV/Lausanne laboratory, urine specimens were inoculated on ORI only and imaged once at 18–24 h. Similarly, MAC plates, which support the growth of fast-growing Gram-negative bacilli, are also read only once at 18–24 h. For ORI plates, only eight FN or three FN results were predicted out of 836 plates at 18 or 24 h, respectively (Fig. S3 and S4). For the MAC plates, only one FN was predicted at 18 h and none at 24 h out of 834 plates (Fig. S5). Bacterial colonies were not identified as image acquisitions and human readings were distant from several months.

### False-positive predictions

Interestingly, among the 1,681 FP calls out of the 49,972 timepoints, 405 (23.5%) and 87 (4.9%) showed a difference of −2 and −4 h, respectively, between the prediction and the first human truth (Fig. S6A and B). This means that approximately one-third of the FP generated by the AI are likely early predictions that are later identified by the human. Re-examination of the 138 urogenital FP plates, which presented the highest rate of FP AI predictions, showed an over-representation of small bacterial growth that generated discrepancies between the AI and the human truth. Most of the cases (63.2%) could be re-categorized by an additional human review as early detection of actual growth by the AI (Fig. S6C). For the CHOC media, re-examination of the 288 plates with FP predictions showed that, again, 48.3% of the FP corresponded to an early detection of growth by the algorithm while the remaining plates showed significant discrepancies with the human truth. Interestingly here, the FP plates presented significantly more small defects within the agar, suggesting that low-quality manufactured plates would negatively impact the prediction (Fig. S6D).

The AI is trained to classify candidates as CFU and to report a plate growth confidence from the identified CFUs with a threshold set at 0.5 (<0.5 no growth, ≥0.5 growth) (Fig. 2B). Although most of the TP prediction showed a probability GROWTH of 1, the FP showed a more dispersed distribution and the CHOC plates accumulated with a low probability growth (Fig. 2C and D). Here, adjusting the algorithm using specific probability thresholds would be possible and especially useful at early timepoints to minimize the FP calls.

### Non-monotonic predictions

**Fig 3:**
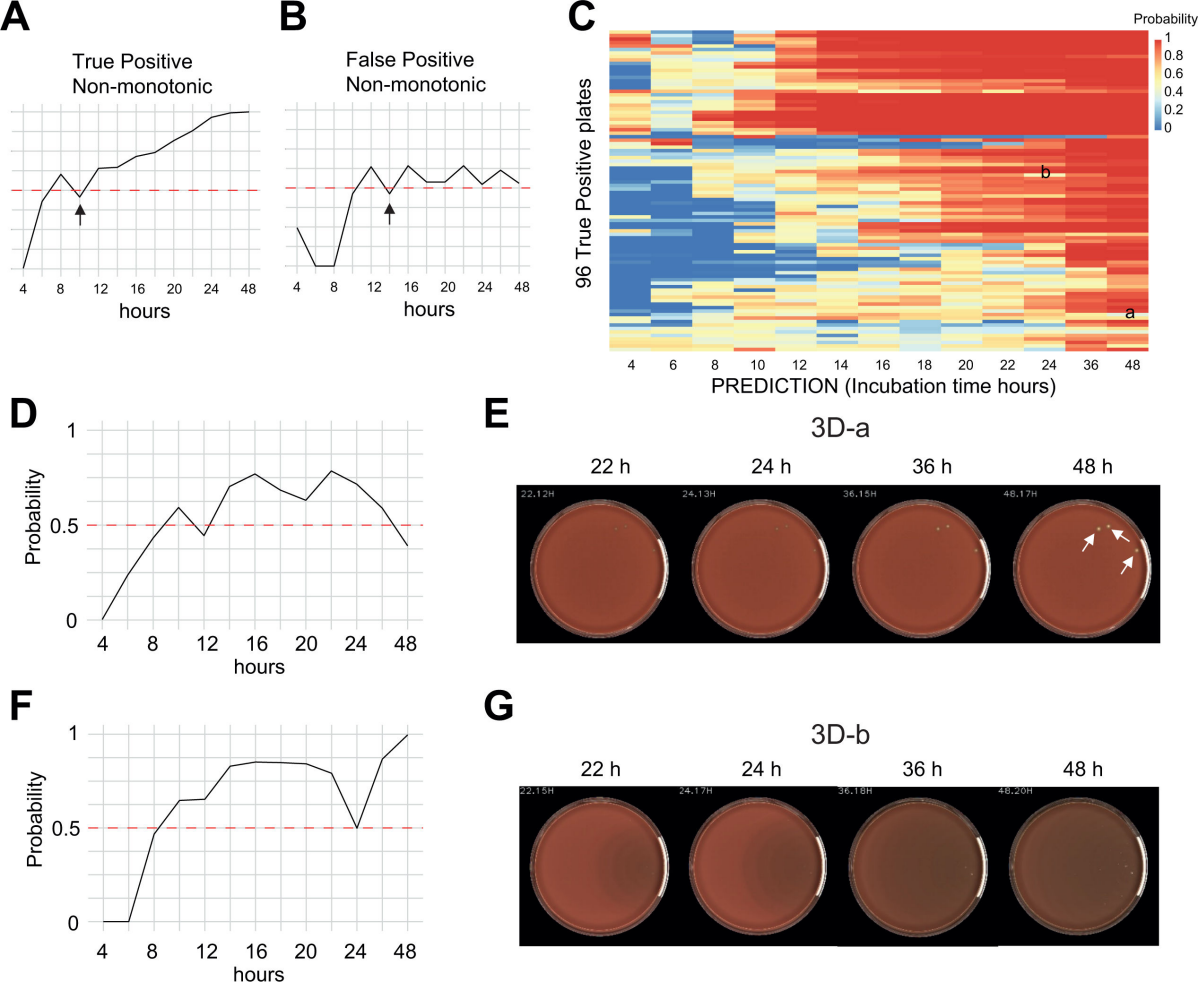
(A and B) Typical profiles for a TP non-monotonic prediction and a FP prediction, respectively. (C) Probability matrix of the 96 non-monotonic predictions with a human truth growth. Most of the plates showed a typical TP non-monotonic prediction profile with a probability close to 1 at 48 h. Two plates, a and b, showed unexpected behaviors. (D and E) Probability plot of the prediction and images of the plate 3D-a showing a decrease of the probability despite the progressive appearance of three well-marked colonies. (F and G) Unexpected drop of the AI growth prediction at 24 h for the plate 3D-b. The swarming waves are not detected by the AI in the absence of a CFU.

As growth is predicted independently at every timepoint, non-monotonic (a growth followed by a no growth statement) behavior of the predictions is possible especially for difficult-to-read plates when growth confidence fluctuates around the positivity threshold. Out of the 3,844 plates, 312 showed a non-monotonic behavior (Fig. S7). Importantly, most of them showed one non-monotonic event (69.8%, Table S5) and only one showed five. This corresponds to either a non-monotonic event at early timepoints corresponding to the detection of a low biomass close to the positivity threshold (96 plates out of 312) (Fig. 3A) or FP calls linked to plate artefacts (216 plates out of 312) (Fig. 3B). Here, the probability remains either slightly below or slightly above the positivity threshold of 0.5. As non-monotonic behavior will occur, an optional sanity check will be included in the final version of the product to prevent the algorithm from releasing a predicted sterile plate if a growth is detected at an earlier timepoint. However, out of the 96 plates with non-monotonic event(s) and a final human truth growth, only two plates showed puzzling behaviors (Fig. 3C, a and b; Table S6), while the remaining ones showed profiles equivalent to the example provided in Figure 3A, with a confident prediction of growth at 48 h (probability close to 1). Therefore, non-monotonic behaviors are not predicted to negatively impact the performance of the system as they will mostly represent FP calls.

### Unexpected predictions

As mentioned above, two plates showed unexpected non-monotonic behaviors. For the plate 3C-a, the probability plot showed that growth was consistently predicted from 14 to 24 h but rather unexpectedly, the probability decreased at 36 and 48 h despite the progressive appearance of three well-defined alpha hemolytic colonies (Fig. 3D and E). This plate was not included in the FN at 48 h due to correct multiple predictions at earlier timepoints. Of relevance here, the AI has been trained using incubation durations up to 24 h and illustrates that stretching the usage of an AI on significantly different context may lead to surprising results. The second surprising behavior involves a CHOC plate where a no growth was flanked by multiple growth predictions (Fig. 3F and G). This plate presented a swarming pattern that negatively impacted the detection of tiny objects on the plate. Indeed, the algorithm is trained to detect well-defined CFU and not diffuse growth pattern such as swarming. However, here, the swarming of the putative *Proteus* spp. without a CFU is rare, and additional plates restricting the swarming are typically present within a specimen.

### Early growth monitoring

**Fig 4:**
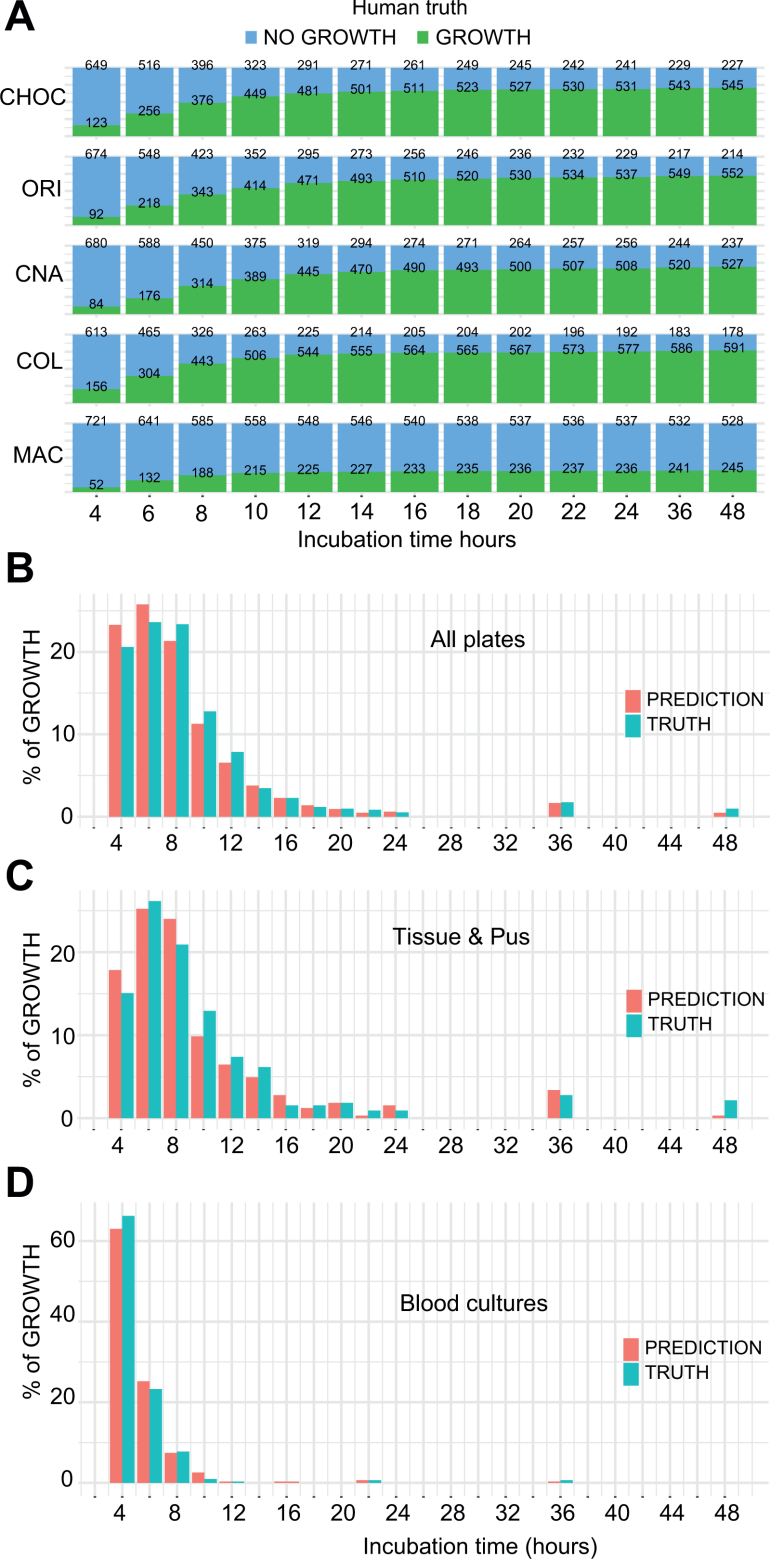
(A) Dynamic of growth (human truth) over time according to media type. growth is stably observed several hours before the usual reading timepoints of 18–24 h. (B–D) The AI efficiently detected growth at early timepoints. Graphs represent the first growth detection by either the human or the AI in (B) all plates, (C) tissue and pus specimens, and (D) blood culture specimens.

Early growth monitoring could be used to (i) prioritize the processing of samples by the microbiologists or to (ii) provide early warning to the clinicians that some bacterial growth is detected. This could be applied to the early detection of Gram-positive cocci in a joint puncture or any bacterial growth in a CSF, for example. Human monitoring of bacterial growth showed that the number of plates with growth reached a plateau several hours before the usual 18–24 h timepoint used in standard microbiology practice (Fig. 4A). Both human and AI could detect growth at early timepoints (Fig. 4B through D). Among the plates with bacterial growth, more than 70% were already detected at 8 h (Fig. 4B; Table S7). By focusing only on the tissue and pus specimens, growth was detected as early as 6 h in 43% of the positive samples (Fig. 4C), while for positive blood cultures, 63% of the growth was detected as early as 4 h (Fig. 4D).

## DISCUSSION

We evaluated an AI algorithm capable of detecting bacterial growth for the identification and release of sterile plates processed by the automated systems BD Kiestra TLA. As this can be performed outside regular laboratory opening hours, it is expected to significantly reduce the turnaround time (TAT). Similarly, but not yet evaluated, Copan recently released the PhenoMATRIX PLUS, designed to automatically segregate positive plates and discard negative ones (24). Previous implementation of imaging applications within the WASPlab or the BD Kiestra TLA systems on either urine specimens or specific screening of bacteria (MRSA, VRE, etc.) already showed a reduction in the TAT and improved laboratory efficiency (15, 25–27).

Here and when available, the AI will report growth or no growth classification along with the growth probability based on 0.5 growth probability threshold. The system will automatically send results to the Laboratory Information System (LIS) either for a specific plate at a specific timepoint or for the specimen when all plates are found sterile at the end of the incubation process. The user will be able to set custom probability thresholds for growth or no growth classification depending on the specimen type or reading timepoint. Lower than 0.5 probability threshold can be used to increase growth detection sensitivity at the expense of decreasing specificity and vice versa. For example, samples subjected to an early growth monitoring with multiple images at low biomass timepoints might be affected by non-monotonic predictions. Increasing the probability threshold could mitigate the problem but at the expense of sensitivity.

Overall, we observed an excellent performance of the AI with a sensitivity of 99.80% (95% CI: 99.53%–99.99%) with five FN plates generated out of 3,844. FP calls (1,681) were generated by the AI out of 49,972 timepoints, but a significant fraction (30%) could rather be attributed to early growth predictions of the AI. Although we have not manually re-evaluated all the FP calls, the reported specificity of 92.89% is likely an underestimate as illustrated by the additional review of the urogenital specimens. Overall, no differences were observed between the different agar plates except for the CHOC, which, despite the revision of some truths, showed a lower specificity. This is probably due to the smaller CHOC training data set in the original UCA. The current version of the AI appears to have some difficulties detecting faint monolayers or non-classical CFU pattern as these were likely not abundant on the training set composed of urine specimen. In one situation, we observed a decrease of the growth prediction after 24 h despite the presence of growing colonies. The AI was trained using incubation durations up to 24 h and this illustrates that stretching the usage of a trained AI on significantly different context (such as hemolysis pattern and/or media color/opacity change over incubation duration) may lead to unexpected results. This can, however, be improved using dedicated training sets for each identified situation. This also highlights that robust and comprehensive validation of AI is required (28, 29). As the evaluation of the AI is dependent on the quality and exhaustivity of the validation set, we cannot claim here to have comprehensively assessed all situations, and therefore a close supervision of the AI post-implementation is recommended.

In addition, the ability of the AI toward an automated early growth monitoring was investigated. Here, the system could provide not only alert messages to the clinician when growth is detected, but more importantly could allow the microbiologists to prioritize the processing of such samples (25, 30, 31). In addition, bacterial growth from subcultures could also be monitored with such method as illustrated in the blood cultures. However, the present algorithm only detects growth but does not assess if the biomass present would be sufficient to start an antimicrobial susceptibility testing (AST) or perform an identification by matrix-assisted desorption definition time of flight (MALDI-ToF). On the other hand, early growth monitoring might not be relevant to all specimen types, as monitoring of respiratory specimens, for example, might be of limited use. Here, incubation must be long enough to identify, mingled within the flora, potential slow-growing pathogens.

In conclusion, an automated method for (i) identification and release of sterile plate at any pre-defined timepoints and for (ii) an early monitoring of bacterial growth was evaluated. Release of sterile plates has obvious advantages and is clearly anticipated to have an important impact on the TAT; however, the value of an early growth monitoring remains to be investigated. Currently, the bacteriology workflow is a day-to-day sequential process with long overnight incubations. Automation has already reduced the culture reading time, but it could open the possibility to move from this sequential process to a more continuous process in which plates are analyzed as soon as growth is detected.

## Data Availability

All data analyzed during this study are included in this published article and its supplemental material files. Raw data for statistical analyses are available here: df_for_publication.xlsx (figshare.com).

## SUPPLEMENTAL MATERIAL

Supplemental figures and tables jcm.01651-23-s0001.pdf Figures S1 to S7; Tables S1 to S7.

ASM does not own the copyrights to Supplemental Material that may be linked to, or accessed through, an article. The authors have granted ASM a non-exclusive, world-wide license to publish the Supplemental Material files. Please contact the corresponding author directly for reuse.

## References

[1] Antonios K, Croxatto A, Culbreath K. 2021. Current state of laboratory automation in clinical microbiology laboratory. Clin Chem 68:99–114. doi:10.1093/clinchem/hvab242 34969105

[2] Bailey AL, Ledeboer N, Burnham C-AD. 2019. Clinical microbiology is growing up: the total laboratory automation revolution. Clin Chem 65:634–643. doi:10.1373/clinchem.2017.274522 30518664

[3] Moreno-Camacho JL, Calva-Espinosa DY, Leal-Leyva YY, Elizalde-Olivas DC, Campos-Romero A, Alcántar-Fernández J. 2018. Transformation from a conventional clinical microbiology laboratory to full automation. Lab Med 49:e1–e8. doi:10.1093/labmed/lmx079 29253199

[4] Croxatto A, Prod’hom G, Faverjon F, Rochais Y, Greub G. 2016. Laboratory automation in clinical bacteriology: what system to choose? Clin Microbiol Infect 22:217–235. doi:10.1016/j.cmi.2015.09.030 26806135

[5] Greub G, Prod’hom G. 2011. Automation in clinical bacteriology: what system to choose? Clin Microbiol Infect 17:655–660. doi:10.1111/j.1469-0691.2011.03513.x 21521409

[6] Burckhardt I. 2018. Laboratory automation in clinical microbiology. Bioengineering (Basel) 5:102. doi:10.3390/bioengineering5040102 30467275 PMC6315553

[7] Leo S, Cherkaoui A, Renzi G, Schrenzel J. 2020. Mini review: clinical routine microbiology in the era of automation and digital health. Front Cell Infect Microbiol 10:582028. doi:10.3389/fcimb.2020.582028 33330127 PMC7734209

[8] Rhoads DD, Novak SM, Pantanowitz L. 2015. A review of the current state of digital plate reading of cultures in clinical microbiology. J Pathol Inform 6:23. doi:10.4103/2153-3539.157789 26110091 PMC4466785

[9] Signoroni A, Ferrari A, Lombardi S, Savardi M, Fontana S, Culbreath K. 2023. Hierarchical AI enables global interpretation of culture plates in the era of digital microbiology. Nat Commun 14:6874. doi:10.1038/s41467-023-42563-1 37898607 PMC10613199

[10] Alouani DJ, Ransom EM, Jani M, Burnham CA, Rhoads DD, Sadri N. 2022. Deep convolutional neural networks implementation for the analysis of urine culture. Clin Chem 68:574–583. doi:10.1093/clinchem/hvab270 35134116

[11] Croxatto A, Marcelpoil R, Orny C, Morel D, Prod’hom G, Greub G. 2017. Towards automated detection, semi-quantification and identification of microbial growth in clinical bacteriology: a proof of concept. Biomed J 40:317–328. doi:10.1016/j.bj.2017.09.001 29433835 PMC6138813

[12] Dauwalder O, Michel A, Eymard C, Santos K, Chanel L, Luzzati A, Roy-Azcora P, Sauzon JF, Guillaumont M, Girardo P, Fuhrmann C, Lina G, Laurent F, Vandenesch F, Sobas C. 2021. Use of artificial intelligence for tailored routine urine analyses. Clin Microbiol Infect 27:1168. doi:10.1016/j.cmi.2020.09.056 33038526

[13] Uwamino Y, Nagata M, Aoki W, Kato A, Daigo M, Ishihara O, Igari H, Inose R, Hasegawa N, Murata M. 2022. Efficient automated semi-quantitative urine culture analysis via BD urine culture app. Diagn Microbiol Infect Dis 102:115567. doi:10.1016/j.diagmicrobio.2021.115567 34731683

[14] Glasson J, Hill R, Summerford M, Olden D, Papadopoulos F, Young S, Giglio S. 2017. Multicenter evaluation of an image analysis device (APAS): comparison between digital image and traditional plate reading using urine cultures. Ann Lab Med 37:499–504. doi:10.3343/alm.2017.37.6.499 28840987 PMC5587822

[15] Faron ML, Buchan BW, Samra H, Ledeboer NA. 2019. Evaluation of WASPLab software to automatically read chromID CPS elite agar for reporting of urine cultures. J Clin Microbiol 58. doi:10.1128/JCM.00540-19 PMC6935927 31694967

[16] Brenton L, Waters MJ, Stanford T, Giglio S. 2020. Clinical evaluation of the APAS (R) independence: automated imaging and interpretation of urine cultures using artificial intelligence with composite reference standard discrepant resolution. J Microbiol Methods 177:106047. doi:10.1016/j.mimet.2020.106047 32920021

[17] Foschi C, Turello G, Lazzarotto T, Ambretti S. 2021. Performance of PhenoMatrix for the detection of group B Streptococcus from recto-vaginal swabs. Diagn Microbiol Infect Dis 101:115427. doi:10.1016/j.diagmicrobio.2021.115427 34120035

[18] Baker J, Timm K, Faron M, Ledeboer N, Culbreath K. 2020. Digital image analysis for the detection of group B Streptococcus from ChromID Strepto B medium using phenomatrix algorithms. J Clin Microbiol 59:e01902-19. doi:10.1128/JCM.01902-19 33087433 PMC7771474

[19] Cherkaoui A, Renzi G, Charretier Y, Blanc DS, Vuilleumier N, Schrenzel J. 2019. Automated incubation and digital image analysis of chromogenic media using copan WASPLab enables rapid detection of vancomycin-resistant enterococcus. Front Cell Infect Microbiol 9:379. doi:10.3389/fcimb.2019.00379 31781516 PMC6851235

[20] Faron ML, Buchan BW, Coon C, Liebregts T, van Bree A, Jansz AR, Soucy G, Korver J, Ledeboer NA. 2016. Automatic digital analysis of chromogenic media for vancomycin-resistant- enterococcus screens using copan WASPLab. J Clin Microbiol 54:2464–2469. doi:10.1128/JCM.01040-16 27413193 PMC5035414

[21] Faron ML, Buchan BW, Vismara C, Lacchini C, Bielli A, Gesu G, Liebregts T, van Bree A, Jansz A, Soucy G, Korver J, Ledeboer NA. 2016. Automated scoring of chromogenic media for detection of methicillin-resistant Staphylococcus aureus by use of WASPLab image analysis software. J Clin Microbiol 54:620–624. doi:10.1128/JCM.02778-15 26719443 PMC4767952

[22] Gammel N, Ross TL, Lewis S, Olson M, Henciak S, Harris R, Hanlon A, Carroll KC. 2021. Comparison of an automated plate assessment system (APAS independence) and artificial intelligence (AI) to manual plate reading of methicillin-resistant and methicillin-susceptible Staphylococcus aureus chromagar surveillance cultures. J Clin Microbiol 59:e0097121. doi:10.1128/JCM.00971-21 34379525 PMC8525556

[23] Van TT, Mata K, Dien Bard J. 2019. Automated detection of Streptococcus pyogenes pharyngitis by use of colorex strep A CHROMagar and WASPLab artificial intelligence chromogenic detection module software. J Clin Microbiol 57:e00811-19. doi:10.1128/JCM.00811-19 31434725 PMC6812993

[24] COPAN ITALIA. 2024. Introducing PhenoMATRIX PLUS. Available from: https://www.copangroup.com/introducing-phenomatrix-plus-2

[25] Cherkaoui A, Renzi G, Vuilleumier N, Schrenzel J. 2019. Copan WASPLab automation significantly reduces incubation times and allows earlier culture readings. Clin Microbiol Infect 25:1430. doi:10.1016/j.cmi.2019.04.001 30986560

[26] Cherkaoui A, Renzi G, Martischang R, Harbarth S, Vuilleumier N, Schrenzel J. 2020. Impact of total laboratory automation on turnaround times for urine cultures and screening specimens for MRSA, ESBL, and VRE carriage: retrospective comparison with manual workflow. Front Cell Infect Microbiol 10:552122. doi:10.3389/fcimb.2020.552122 33194794 PMC7664309

[27] Graham M, Tilson L, Streitberg R, Hamblin J, Korman TM. 2016. Improved standardization and potential for shortened time to results with BD kiestra (TM) total laboratory automation of early urine cultures: a prospective comparison with manual processing. Diagn Microbiol Infect Dis 86:1–4. doi:10.1016/j.diagmicrobio.2016.06.020 27422083

[28] DeYoung B, Morales M, Giglio S. 2022. Microbiology 2.0-A “behind the scenes” consideration for artificial intelligence applications for interpretive culture plate reading in routine diagnostic laboratories. Front Microbiol 13:976068. doi:10.3389/fmicb.2022.976068 35992715 PMC9386241

[29] Glasson J, Hill R, Summerford M, Giglio S. 2016. Observations on variations in manual reading of cultures. J Clin Microbiol 54:2841–2841. doi:10.1128/JCM.01380-16 27582515 PMC5078566

[30] Cherkaoui A, Schrenzel J. 2022. Total laboratory automation for rapid detection and identification of microorganisms and their antimicrobial resistance profiles. Front Cell Infect Microbiol 12:807668. doi:10.3389/fcimb.2022.807668 35186794 PMC8851030

[31] Bailey AL, Burnham CAD. 2019. Reducing the time between inoculation and first-read of urine cultures using total lab automation significantly reduces turn-around-time of positive culture results with minimal loss of first-read sensitivity. Eur J Clin Microbiol Infect Dis 38:1135–1141. doi:10.1007/s10096-019-03512-3 30806903

