# Supplementary material for: Development and evaluation of an artificial intelligence for bacterial growth monitoring in clinical bacteriology
Source: J Clin Microbiol. 2024 Apr 4;62(5):e01651-23. doi: 10.1128/jcm.01651-23 (PMC11077979; doi:10.1128/jcm.01651-23)
Supplement: Supplemental figures and tables — Figures S1 to S7; Tables S1 to S7. [file jcm.01651-23-s0001.pdf]

Supplementary Material

Supplementary figures

Figure S1

A-B. Sensitivity is not media dependent while specificity is significantly lower in CHOC plates. Error bars represent 95% CI. **C-D.** Sensitivity remains constant over specimen type while specificity is lower in urogenital specimens. Error bars represent 95% CI. **E-J**. Examples of the methodology used to generate the confusion matrix presented in Figure 1D. The white diagonal represents perfect predictions between the AI and the human. Teal colored boxes represent the counts generated in the matrix. Panel E represents two FN predictions of the AI before a full agreement at latter timepoints with the human truth while panel F shows two FP predictions and then agreement. Panels G and H show how the FP and FN plots are generated. In panel I, if the reading is scheduled at 16 h for the AI, the system will have a 4 h delay compared to the human (lower red dashed box). If the reading is scheduled at 18 h, the system will have only 2 h delay compared to the human (upper red dashed box). The same logic applies for the FP calls in panel J. If the reading is scheduled at 12 h for the AI, the system will be 4 h early compared to the human (left orange dashed box). If the reading is scheduled at 14 h for the AI, the system will be only 2 h early compared to the human (right orange dashed box).

Figure S2

Images at all timepoints of the 10 FN plates predictions described in Table S4

Figure S3

Images at all timepoints of the 8 FN ORI plates at 18 h.

Figure S4

Images at all timepoints of the 3 FN ORI plates at 24 h.

Figure S5

Images at all timepoints of the single FN MAC plates at 18 h.

Figure S6

A. The same confusion matrix as presented in Figure 1D and Figure S2E-J was generated but using only the first human detection of *GROWTH* as reference. Again here, FP are found above the diagonal and the FN are below, but the counts represent the first human *GROWTH* detection. This allows to count the number of plates with a 2 h early prediction by the AI (light

orange dashed box) or with a 4 h early prediction by the AI (dark orange dashed box). **B.** Example of the methodology used to generate the confusion matrix presented in panel A. **C.** Representative plate of a urogenital specimen showing a discrepancy between the AI and the human. Here the AI detected growth several hours before the human. **D.** FP plates showed an enrichment of artefacts estimated using the difference between the number of CFUs and the number of objects detected by the AI. P-value (Wilcoxon–Mann–Whitney test) was calculated comparing this subpopulation of plates against the TP ones.

Figure S7
Growth predictions over time of the 301 plates showing a non-monotonic event.

Supplementary tables

Table S1
Sensitivity, specificity, and positive and negative predictive values over time. Data are presented with lower and upper 95% CI. Right table represents the number of observations.

Table S2
Sensitivity, specificity, and positive and negative predictive values according to plate media. Data are presented with lower and upper 95% CI. Right table represents the number of observations.

Table S3
Sensitivity, specificity, and positive and negative predictive values according to specimen type. Data are presented with lower and upper 95% CI. Right table represents the number of observations.

Table S4
Detailed data for the 10 FN plates. Light grey are plates with a revised truth as *GROWTH*. Dark grey are plates with a corrected TRUTH for *GROWTH* to *NO GROWTH*.

Table S5
Number of non-monotonic events prediction per plate.

Table S6
Detailed data of plate a and b presented in Figure 3E-H.

Table S7

Cumulative percentages of growth detection at the different timepoint according to media and specimen type described in Figure 4. The first human truth or AI prediction is used respectively.

Figure S1

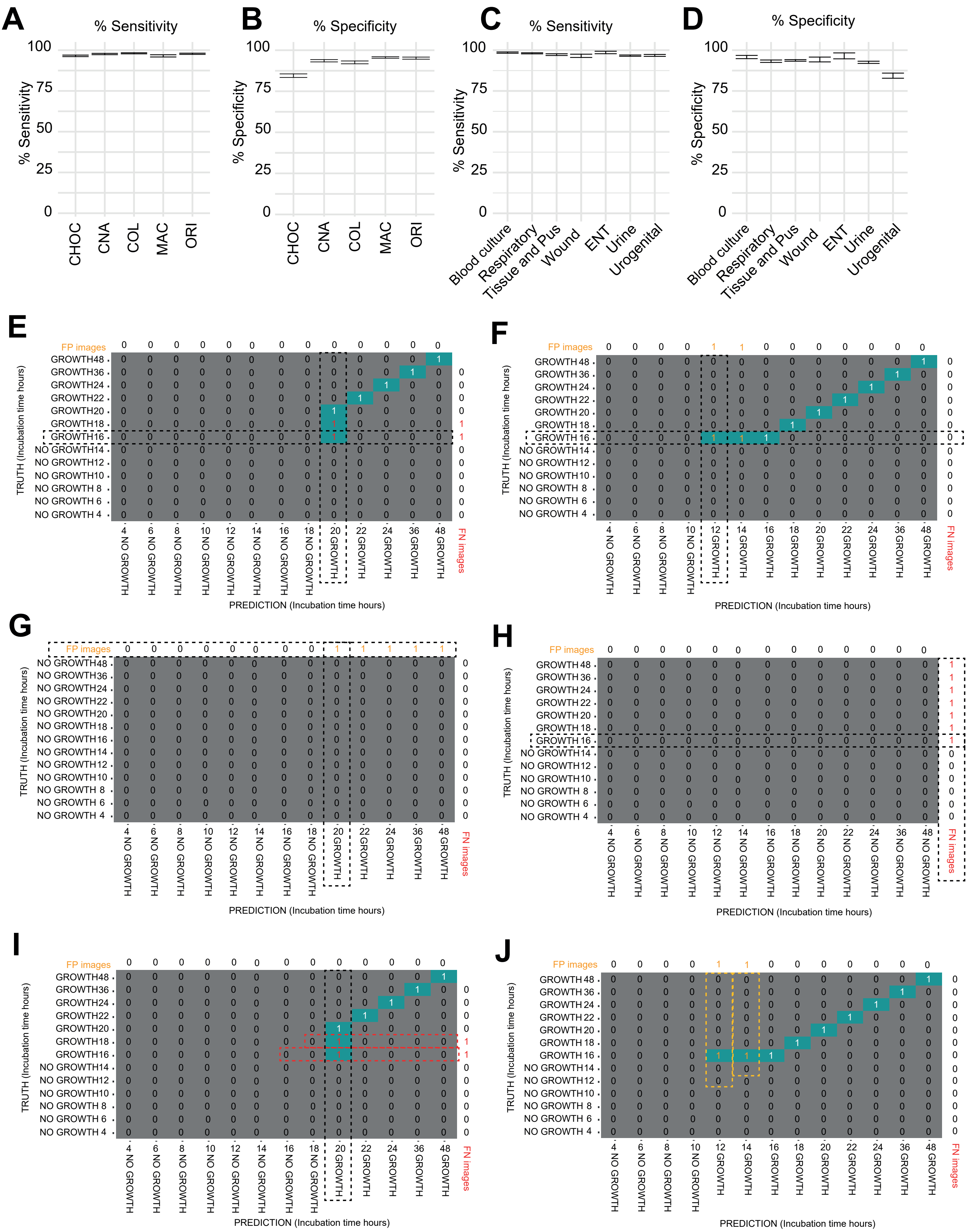
A
% Sensitivity
% Sensitivity
CHOC
CNA
COL
MAC
ORI
B
% Specificity
% Specificity
C
% Sensitivity
Blood culture
Respiratory
Tissue and Pus
Wound
ENT
Urine
Urogenital
D
% Specificity
E
F
G
H
I
J
FP images
FN images
TRUTH (Incubation time hours)
PREDICTION (Incubation time hours)
GROWTH48
GROWTH36
GROWTH24
GROWTH22
GROWTH20
GROWTH18
GROWTH16
NO GROWTH14
NO GROWTH12
NO GROWTH10
NO GROWTH 8
NO GROWTH 6
NO GROWTH 4

Figure S2

CO2-CHOC-2003-T52hhjwamFtbg7h0xP6-2/11

TOP_BLACK

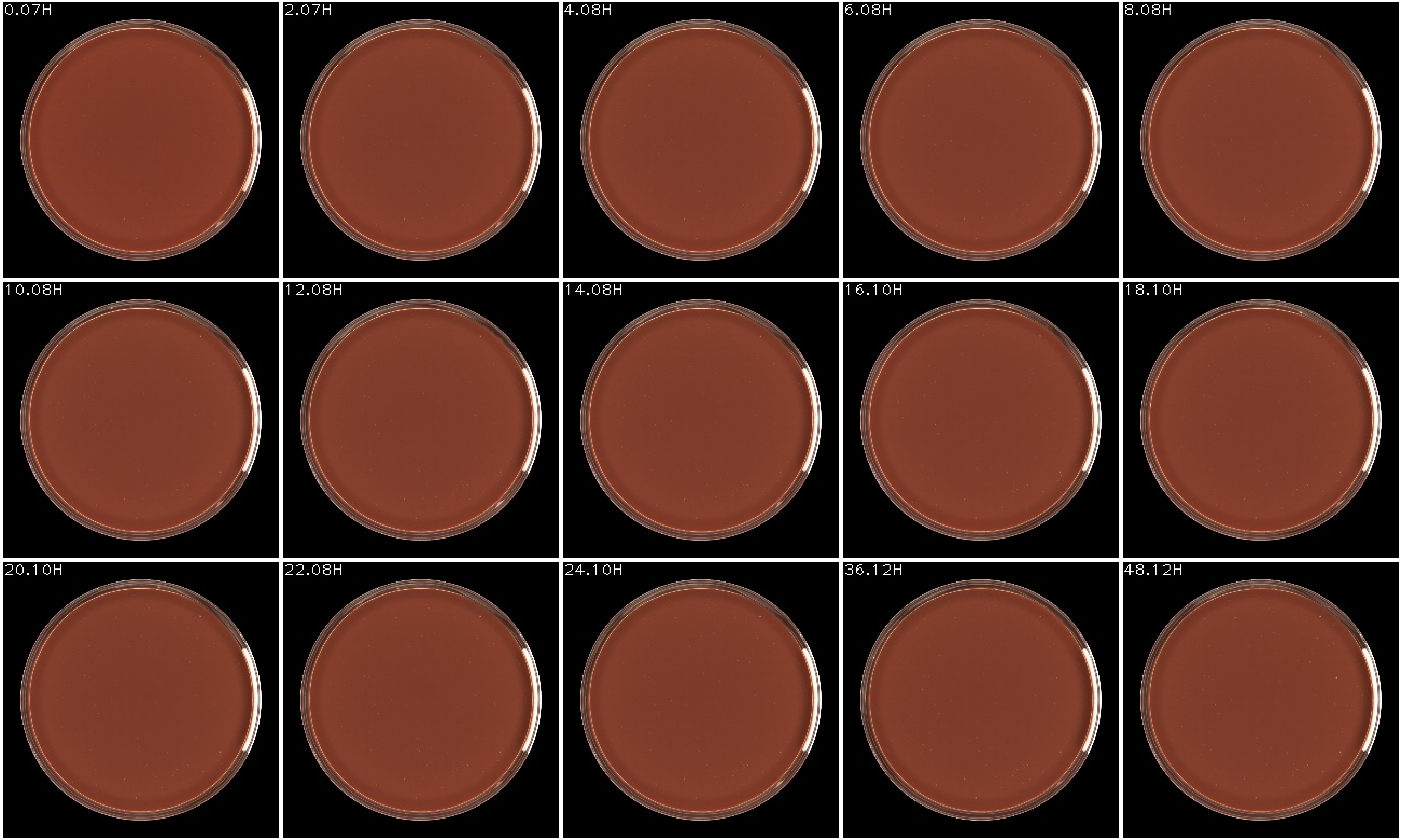

CO2-CHOC-2003-hQcYu0dtCyXRHWsA3wa-3/11

TOP_BLACK

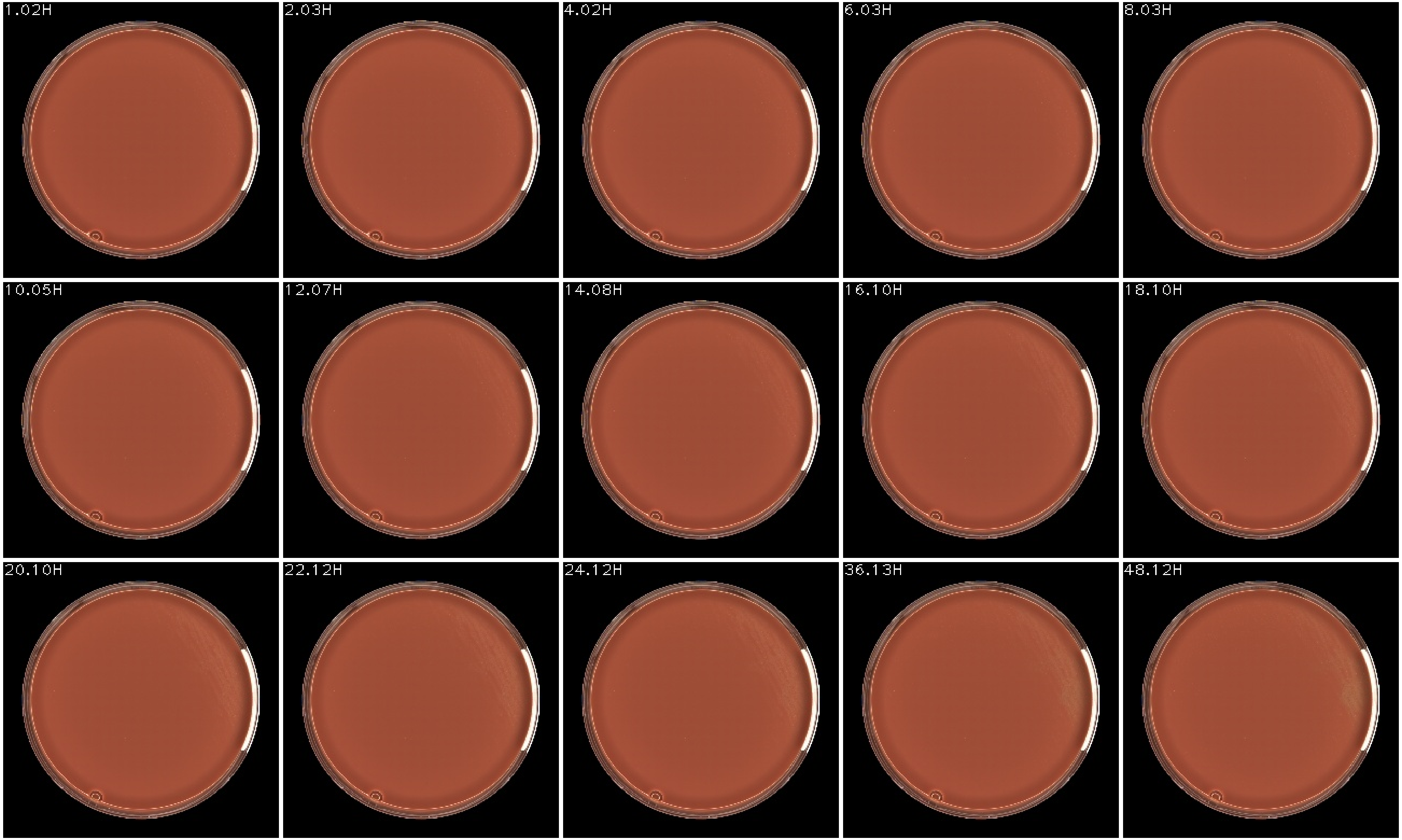

CO2-CHOC-2003-rvVvnqE1L4UwAkCFI16-4/11

TOP_BLACK

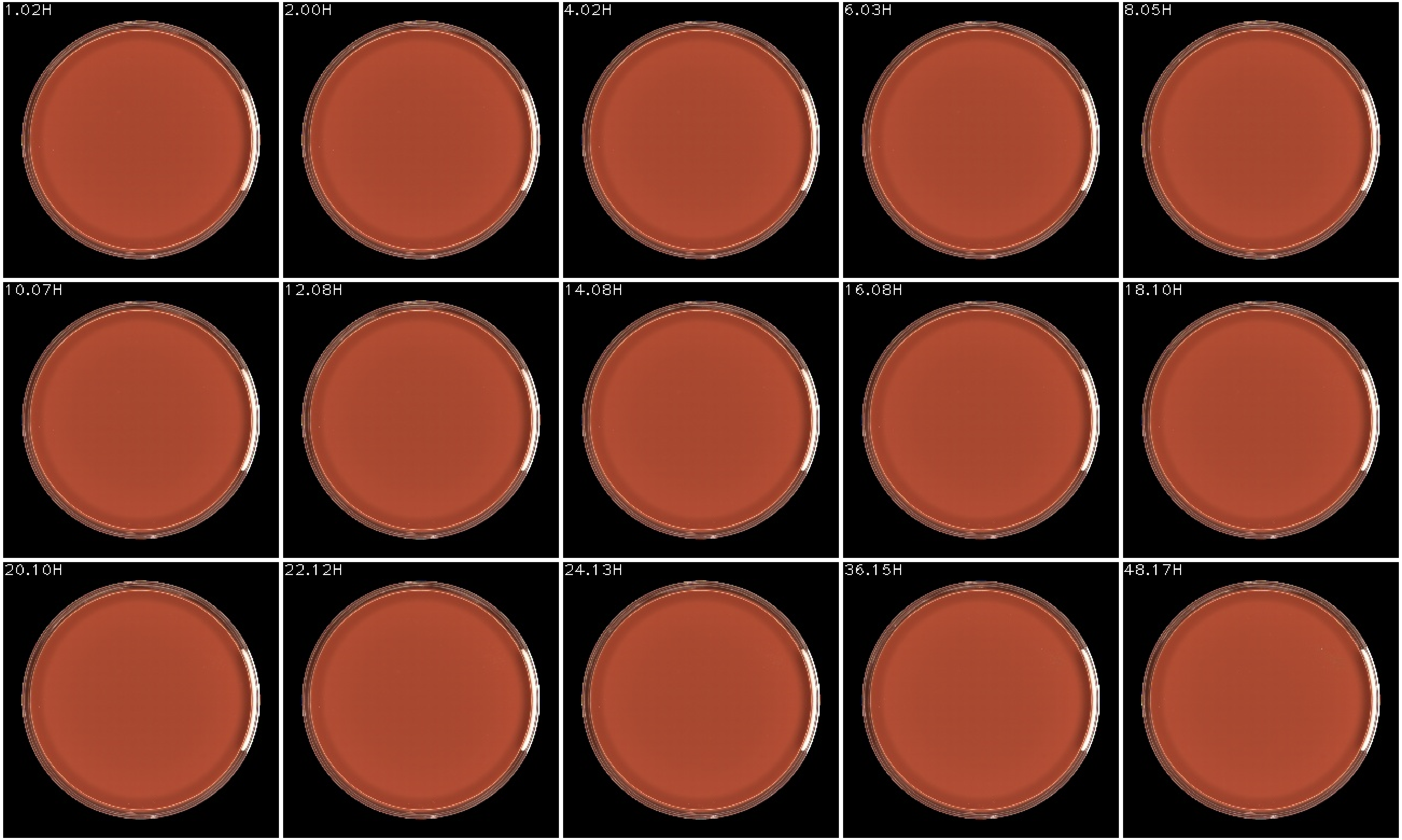

CO2 CNA-2003-3Oc8OLIERvOsKgN8I4D-5/11

TOP_BLACK

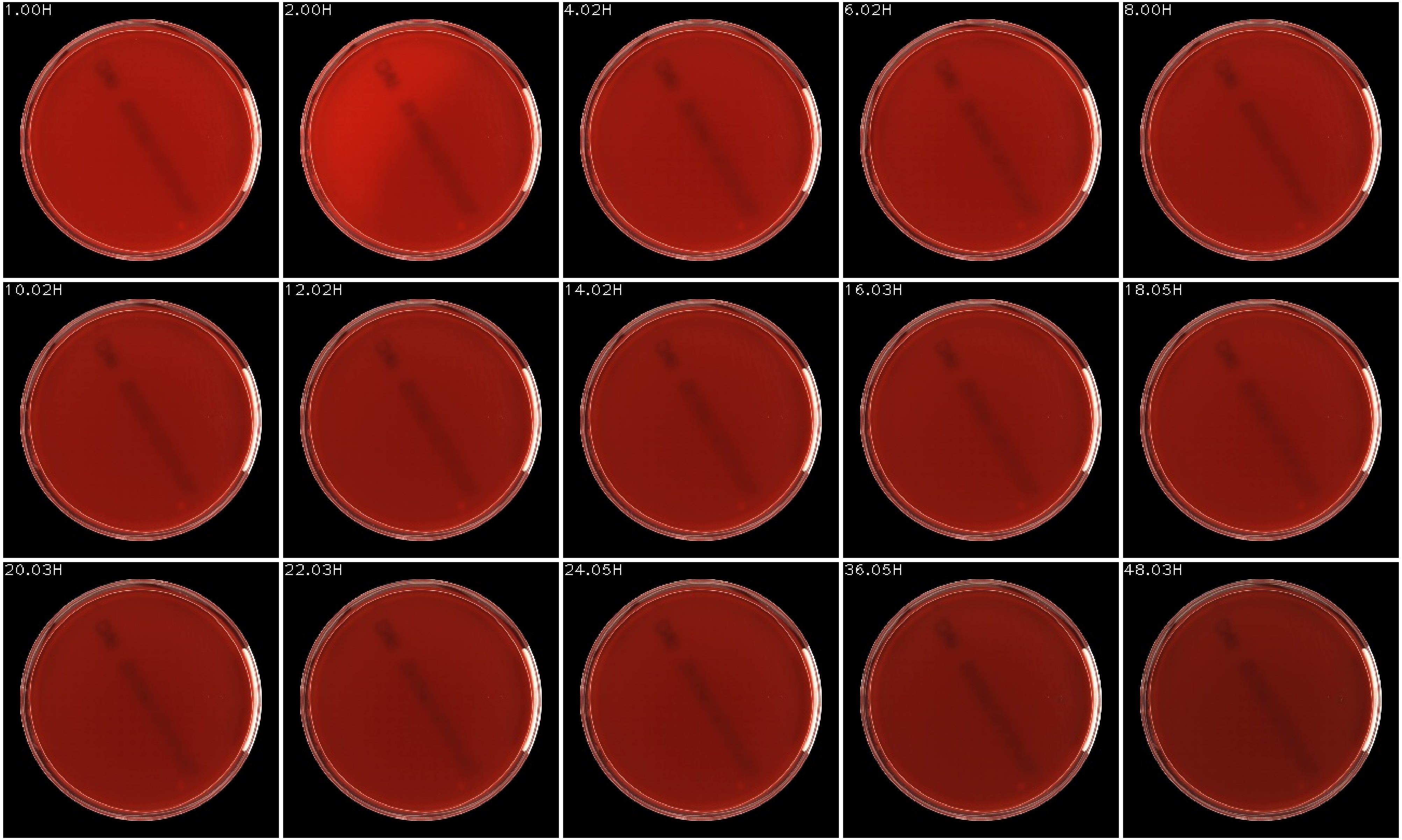

CO2-CNA-2003-bjO1UFnEtLGesY2bhHW-6/11

TOP_BLACK

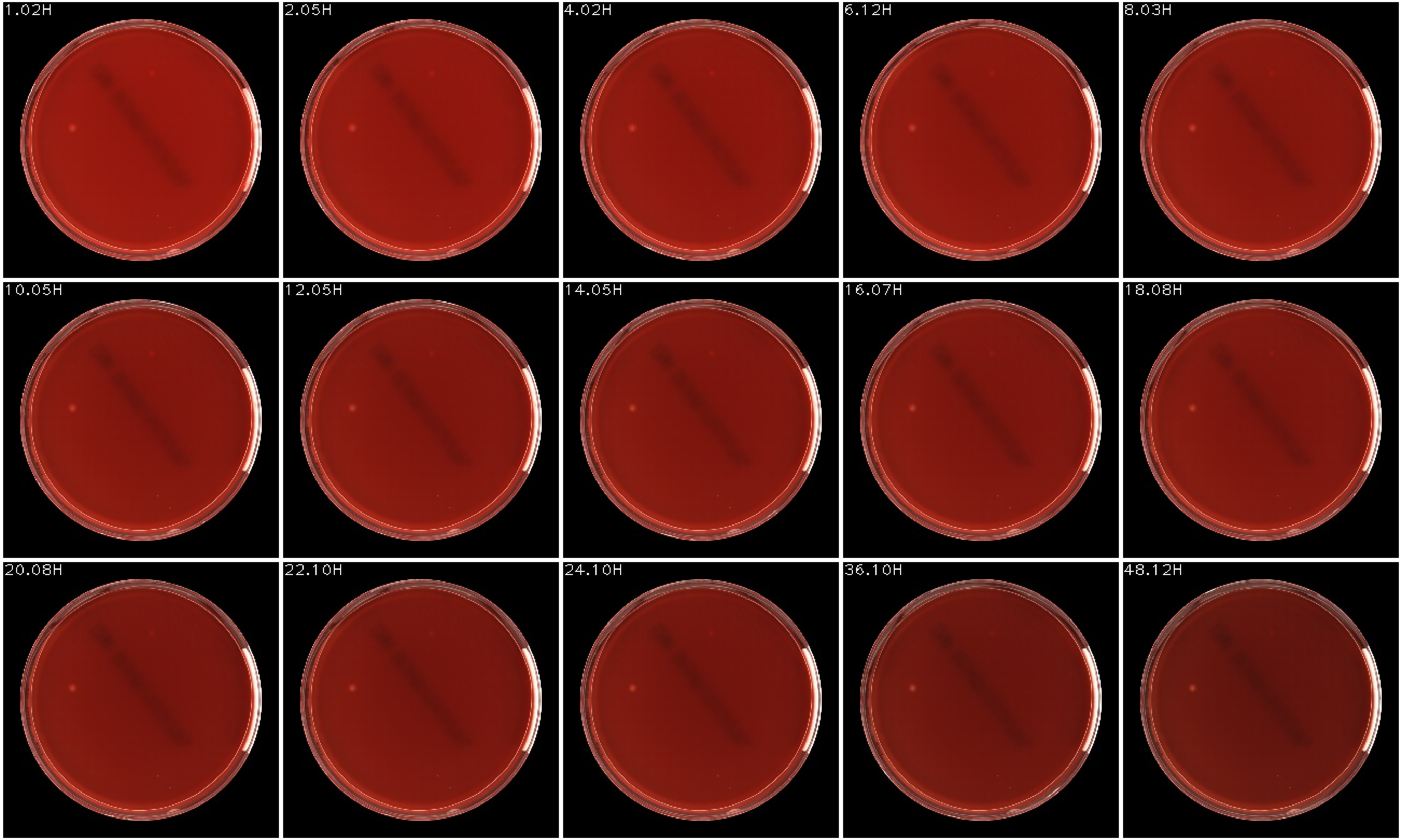

CO2-COL5BP-2003-U5hGx44lsHShVNKcxp6-7/11

TOP_BLACK

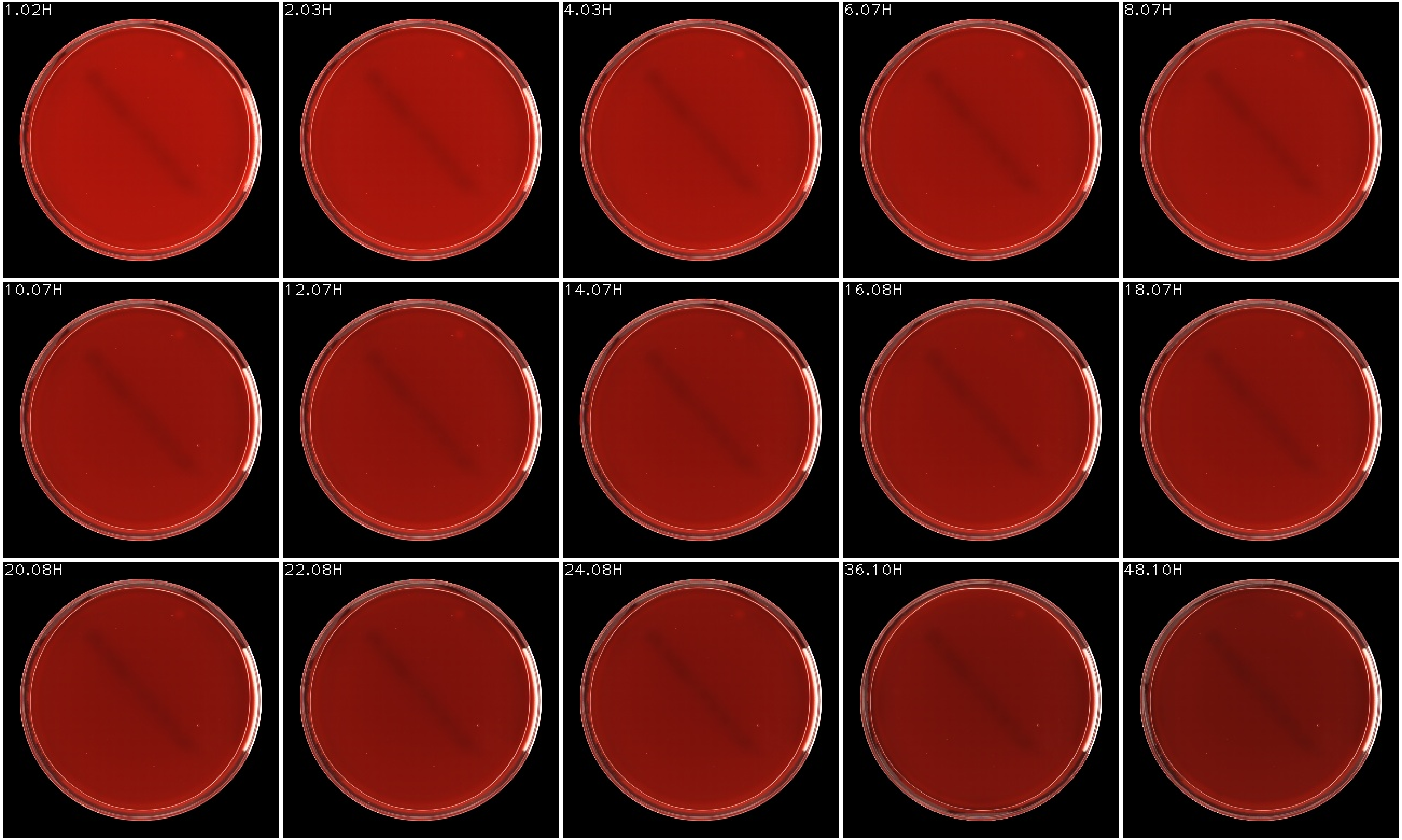

O2-CHROM_ORI-2003-RINL0rP30fpZYPCczY3-8/11

TOP_BLACK

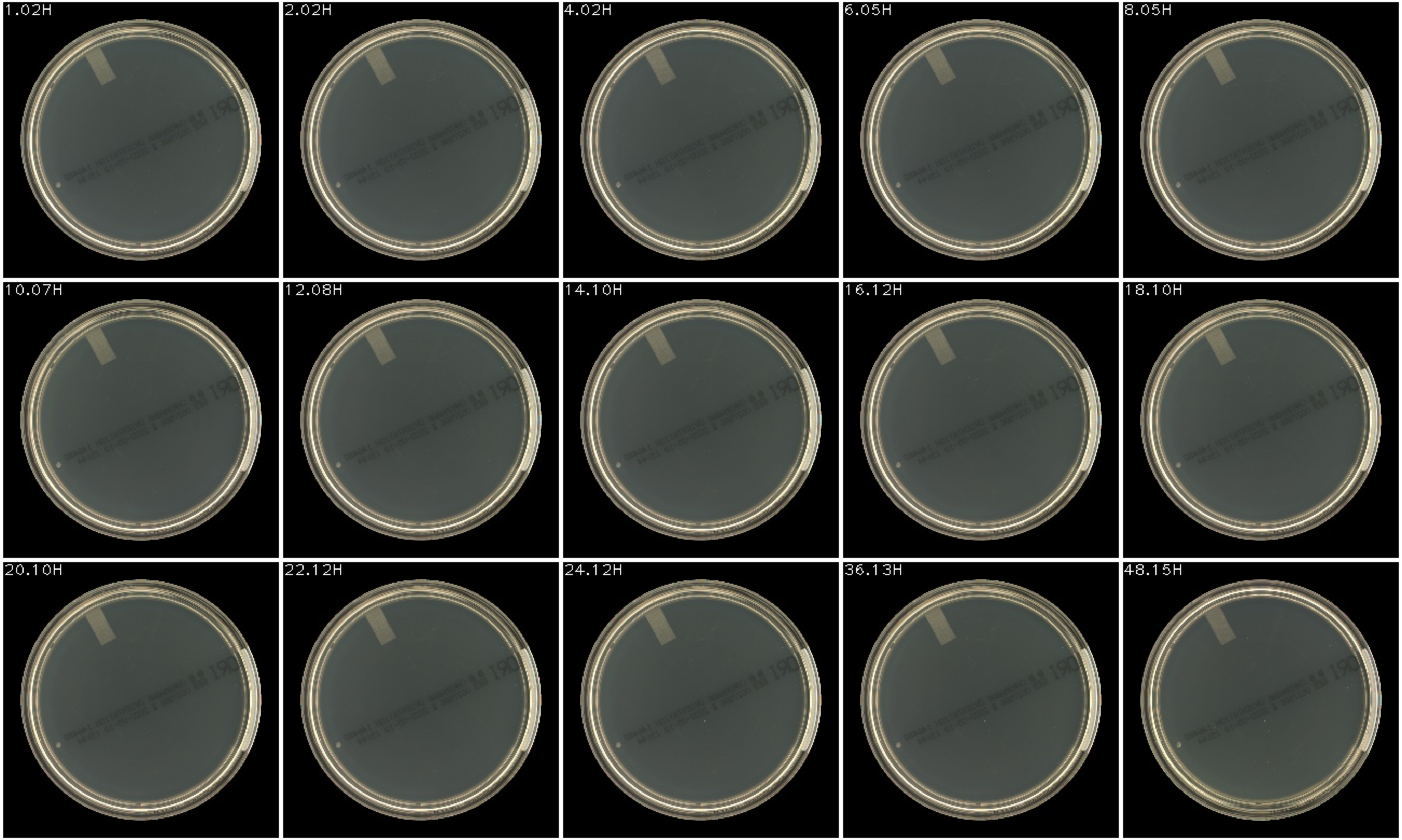

O2-CHROM_ORI-2003-fUCsVZdMk1eAkymRIKK-9/11

TOP_BLACK

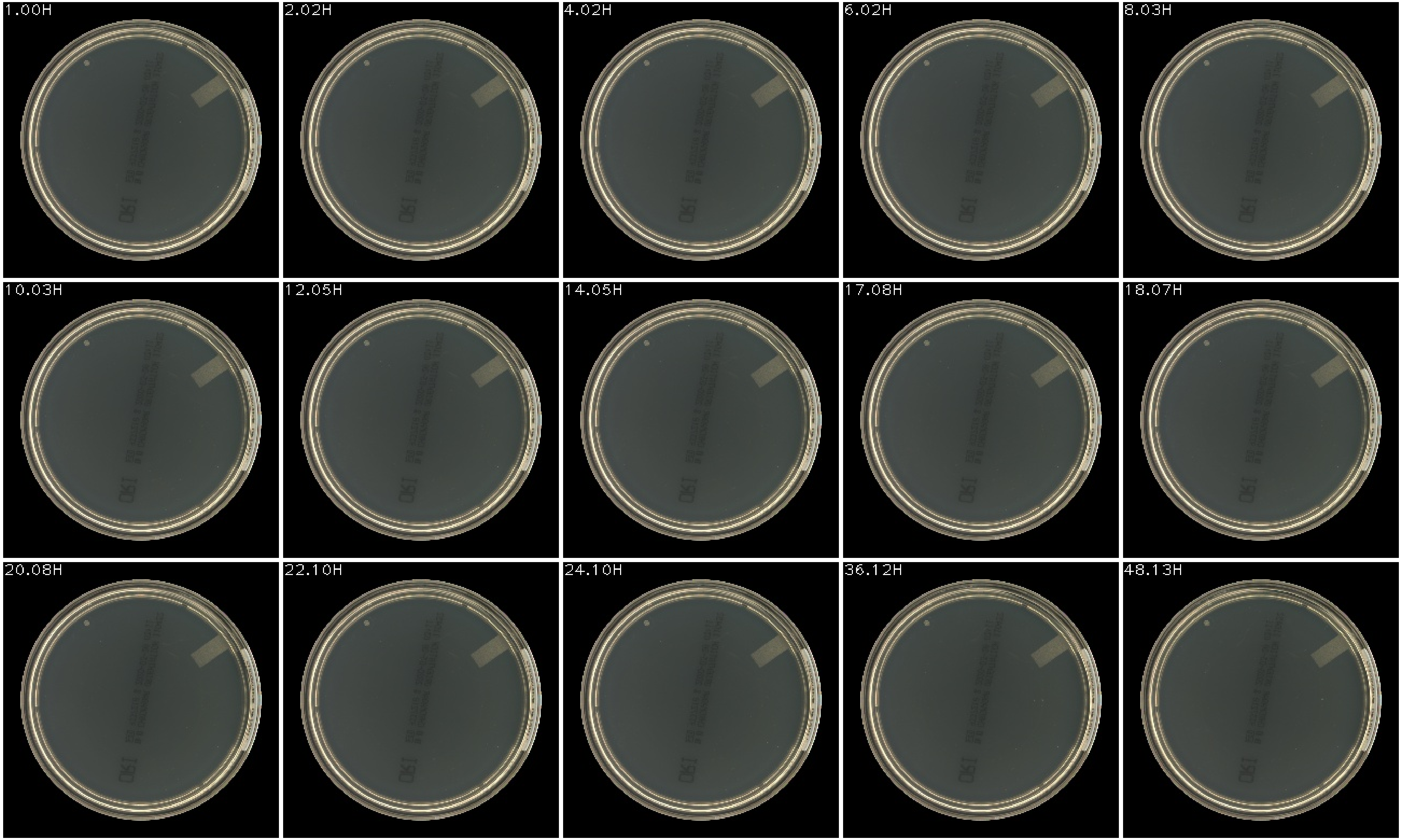

O2-CHROM_ORI-2003-vnuycY5q7qgJpeMLSnk-10/11

TOP_BLACK

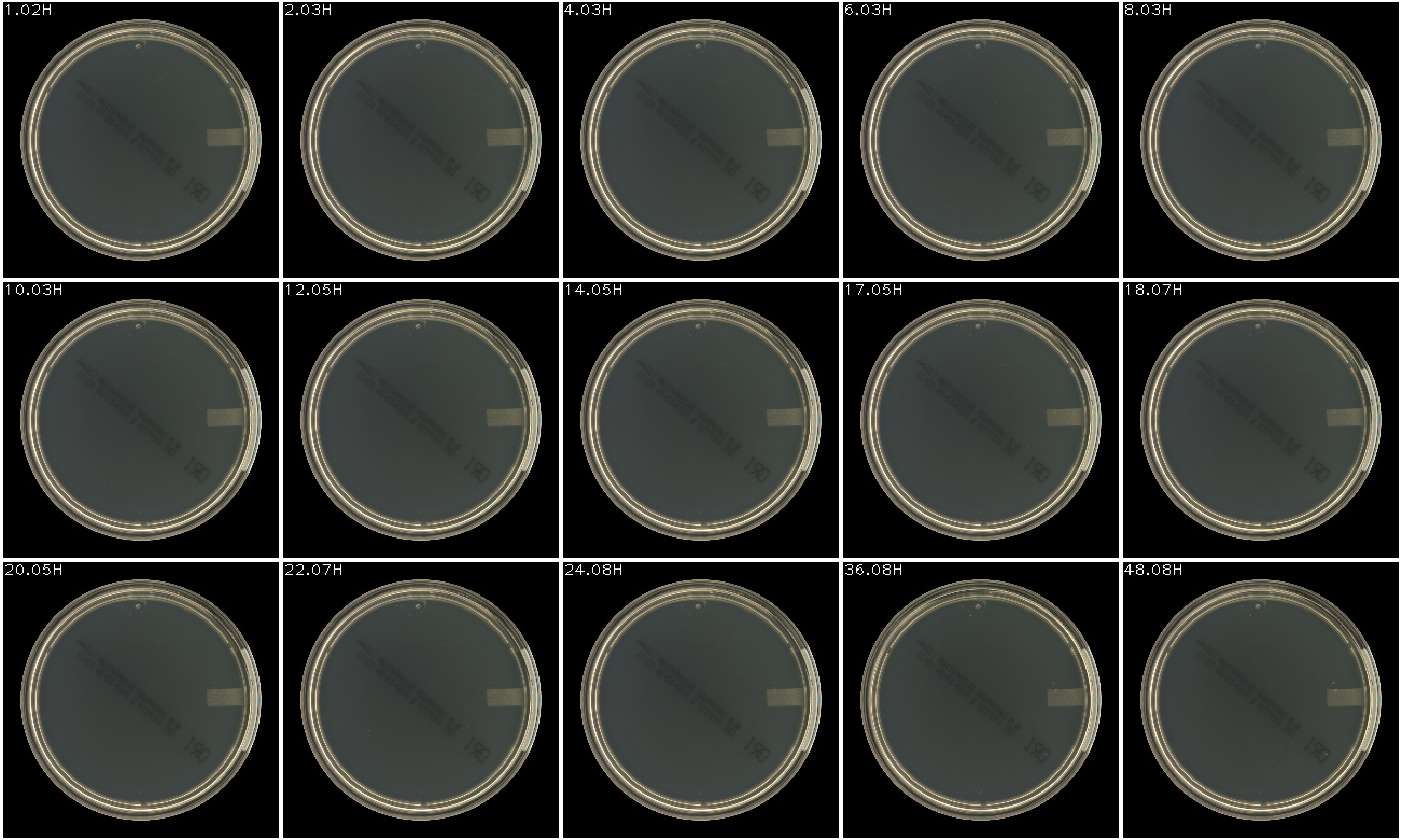

O2-MAC-2003-HmON28saEqhV48Q4lxW-11/11

TOP_BLACK

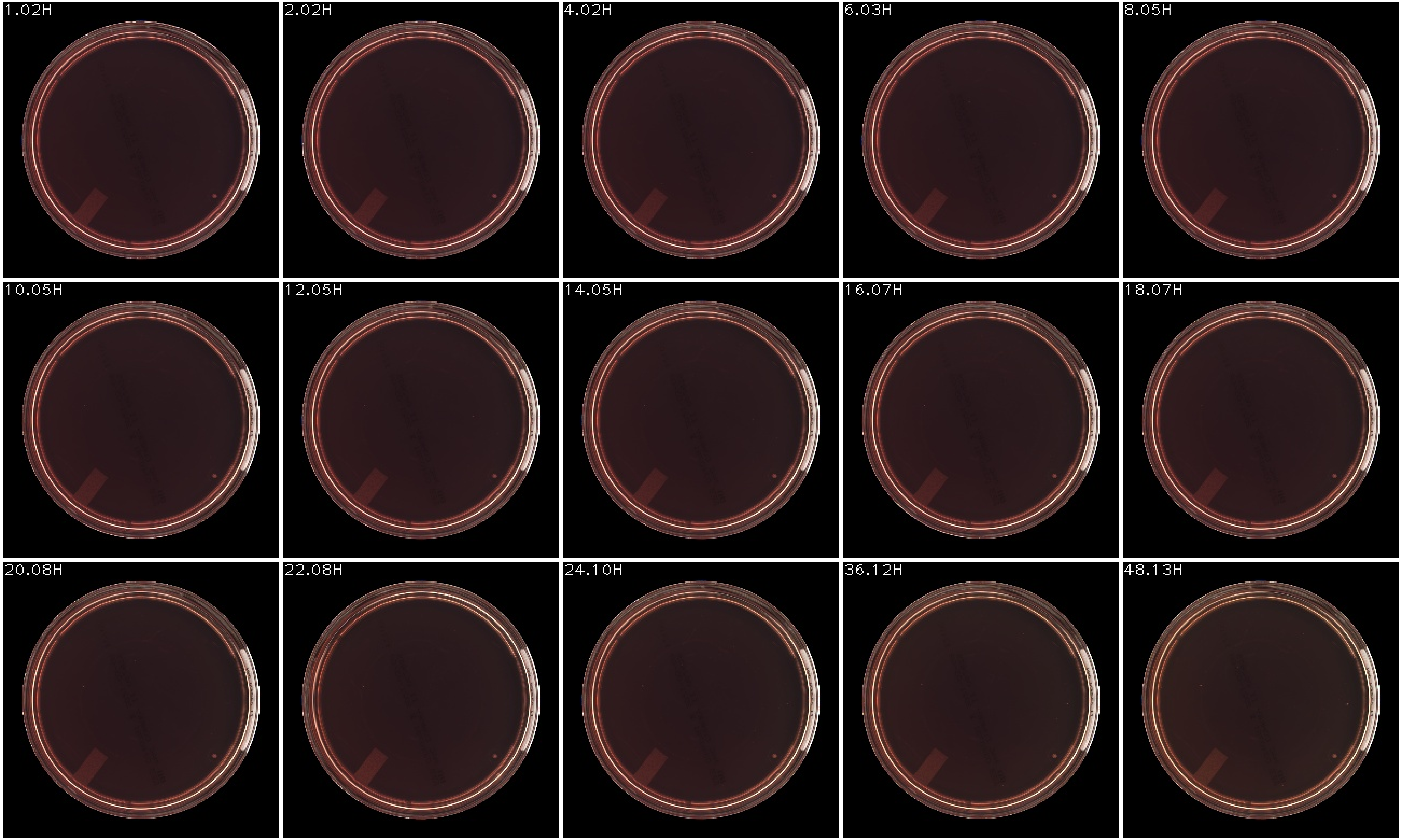

Figure S3

O2-CHROM_ORI-2003-1ig3JAvwFsQ5z38x7z2-1/8

TOP_BLACK

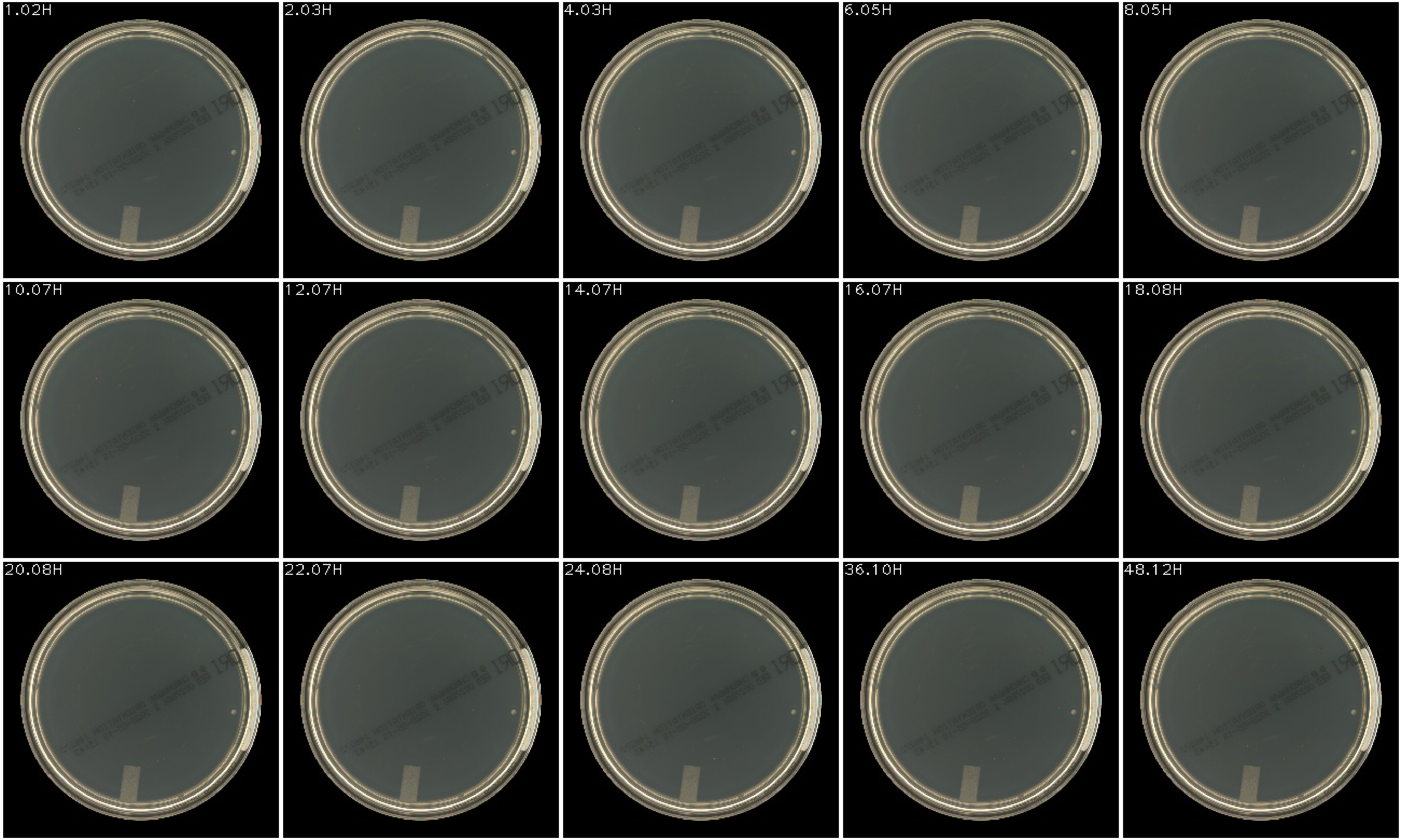

O2-CHROM_ORI-2003-A2PkFJ7BFd67HbAlI0H-2/8

TOP_BLACK

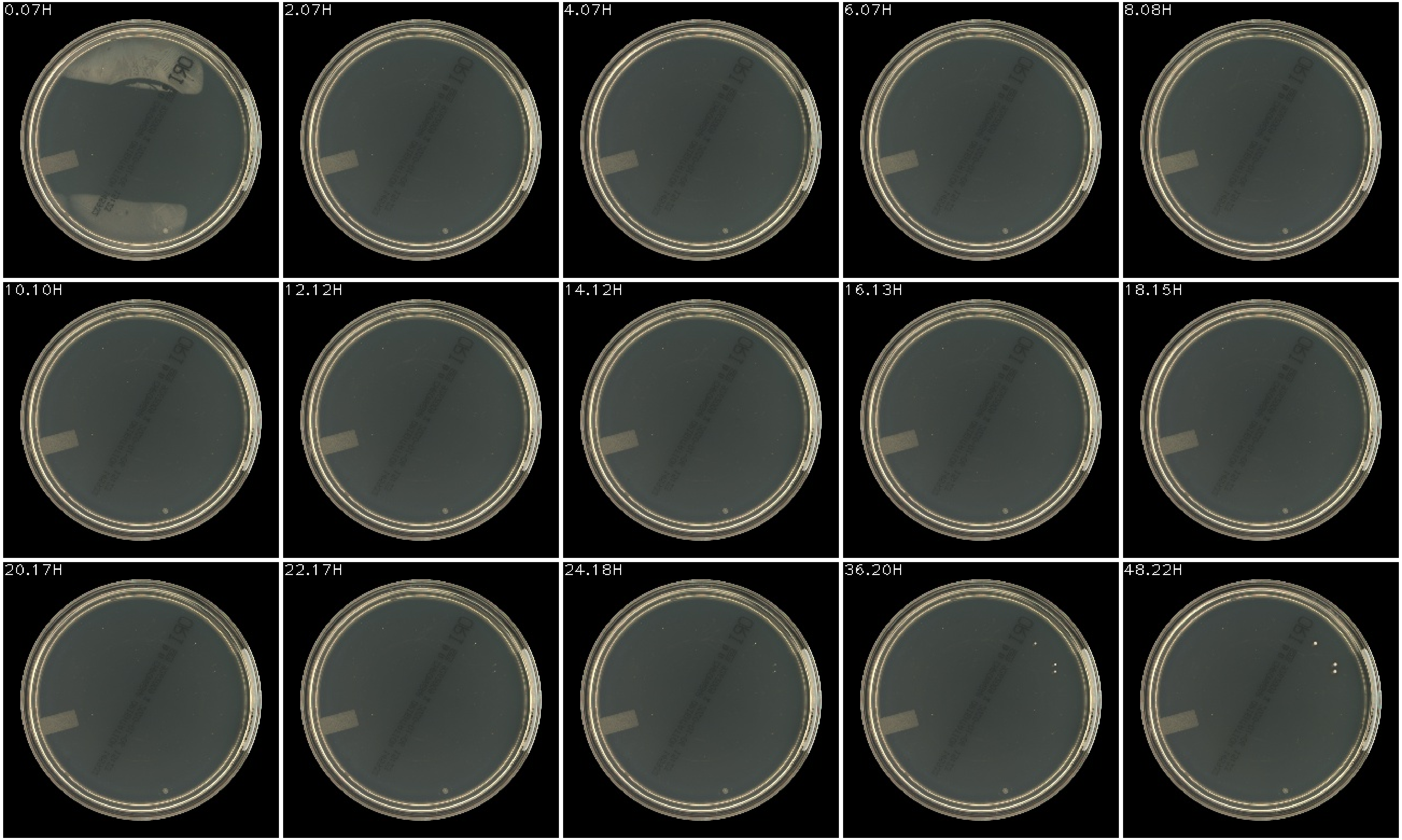

O2-CHROM_ORI-2003-I8huXnNhPZ4SbaX7Aqn-3/8

TOP_BLACK

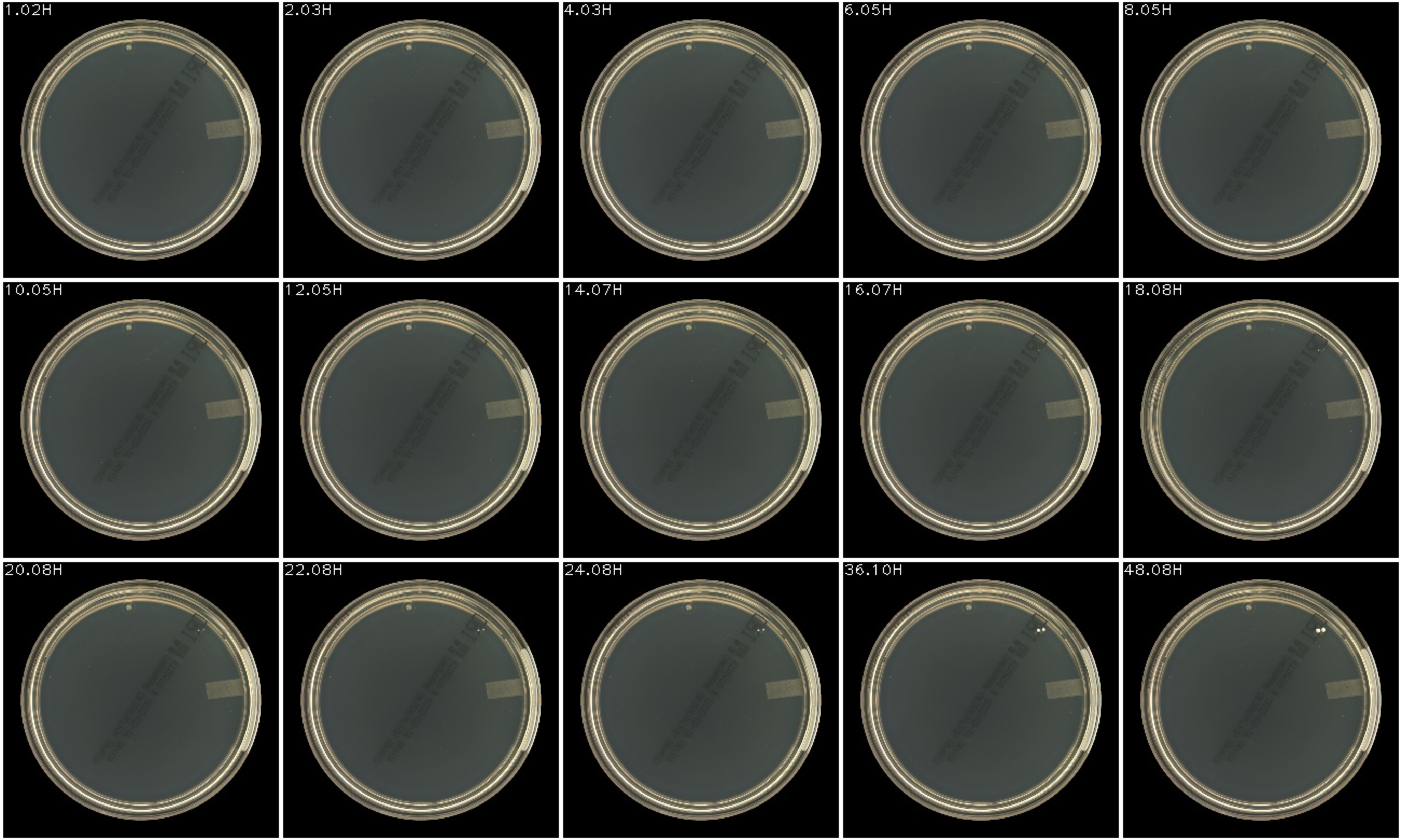

O2-CHROM_ORI-2003-K3ooJPWZ55aGwZ1WdTm-4/8

TOP_BLACK

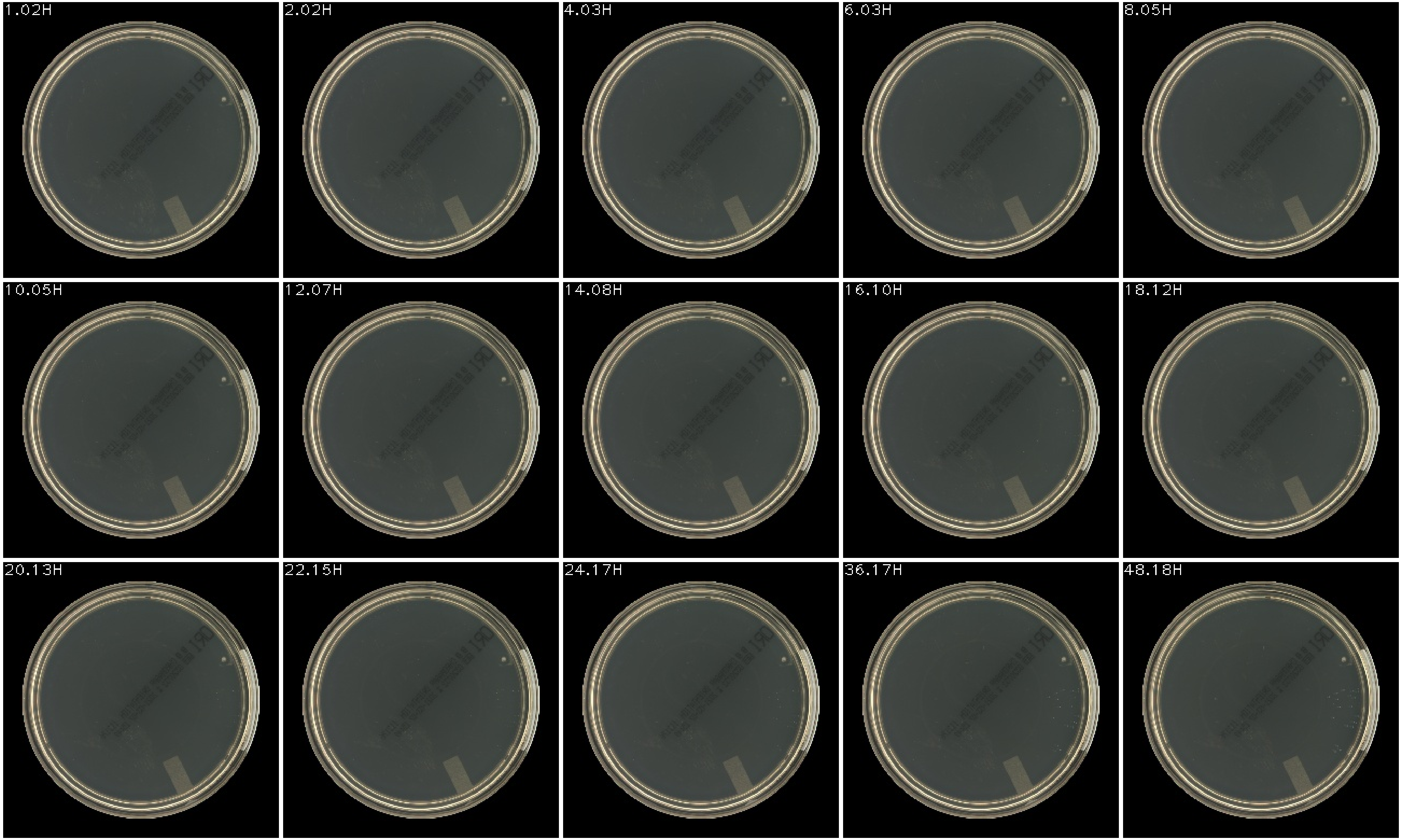

O2-CHROM_ORI-2003-OEF3WJP6a4mLw1xKr1A-5/8

TOP_BLACK

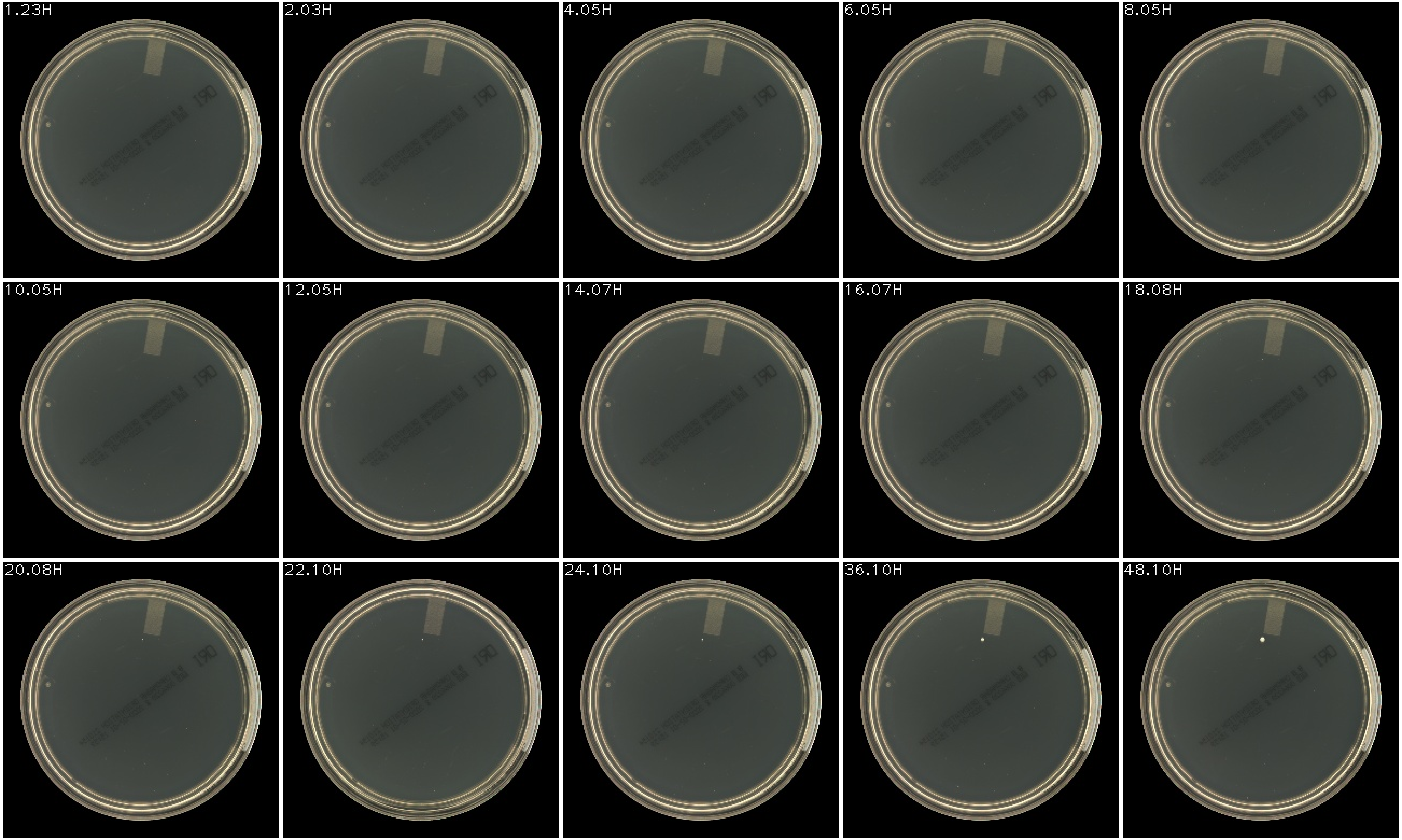

O2-CHROM_ORI-2003-Qe6XlsrGvmHPVol8JL0-6/8

TOP_BLACK

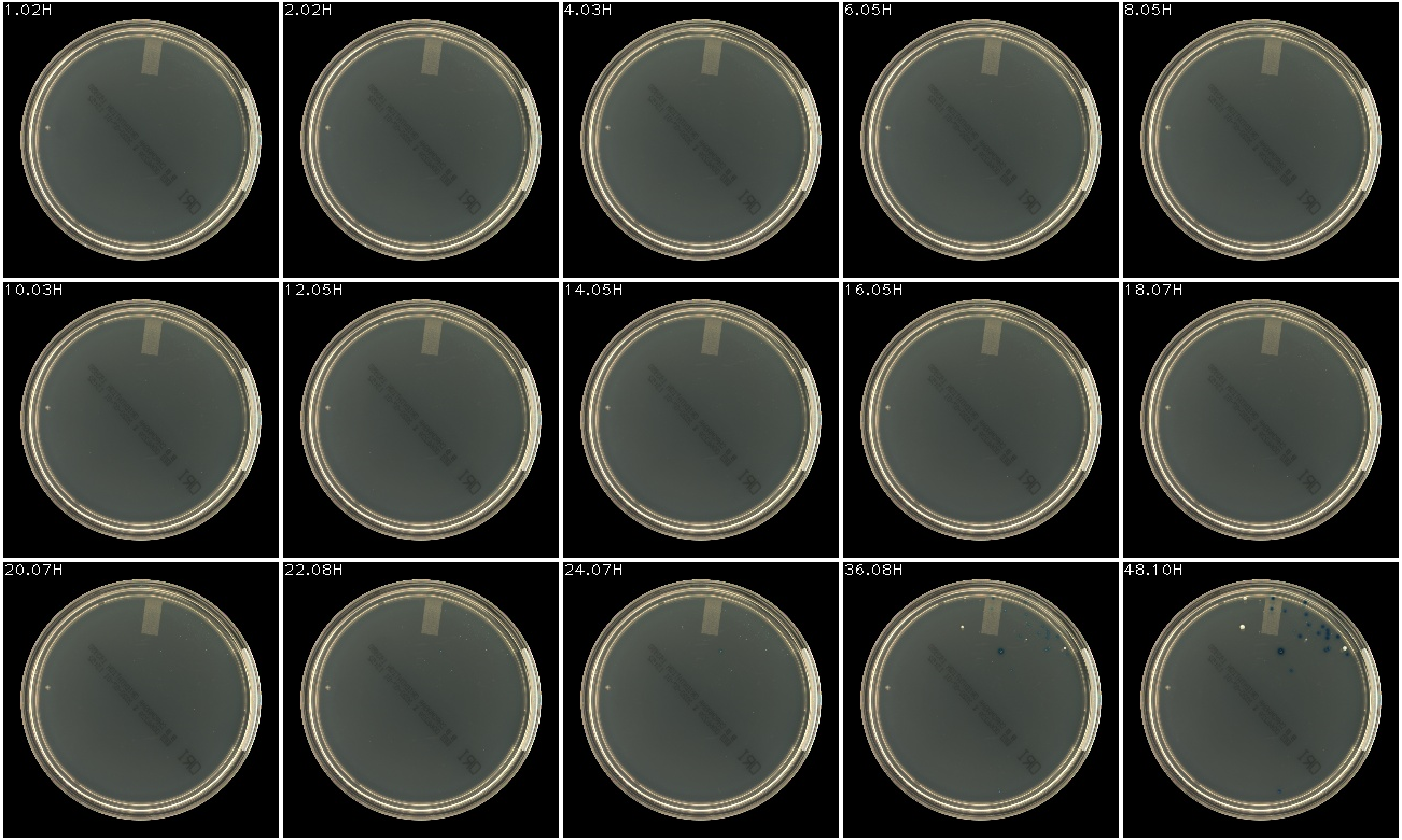

O2-CHROM_ORI-2003-Xzi6unB2Nbq1laOH1bT-7/8

TOP_BLACK

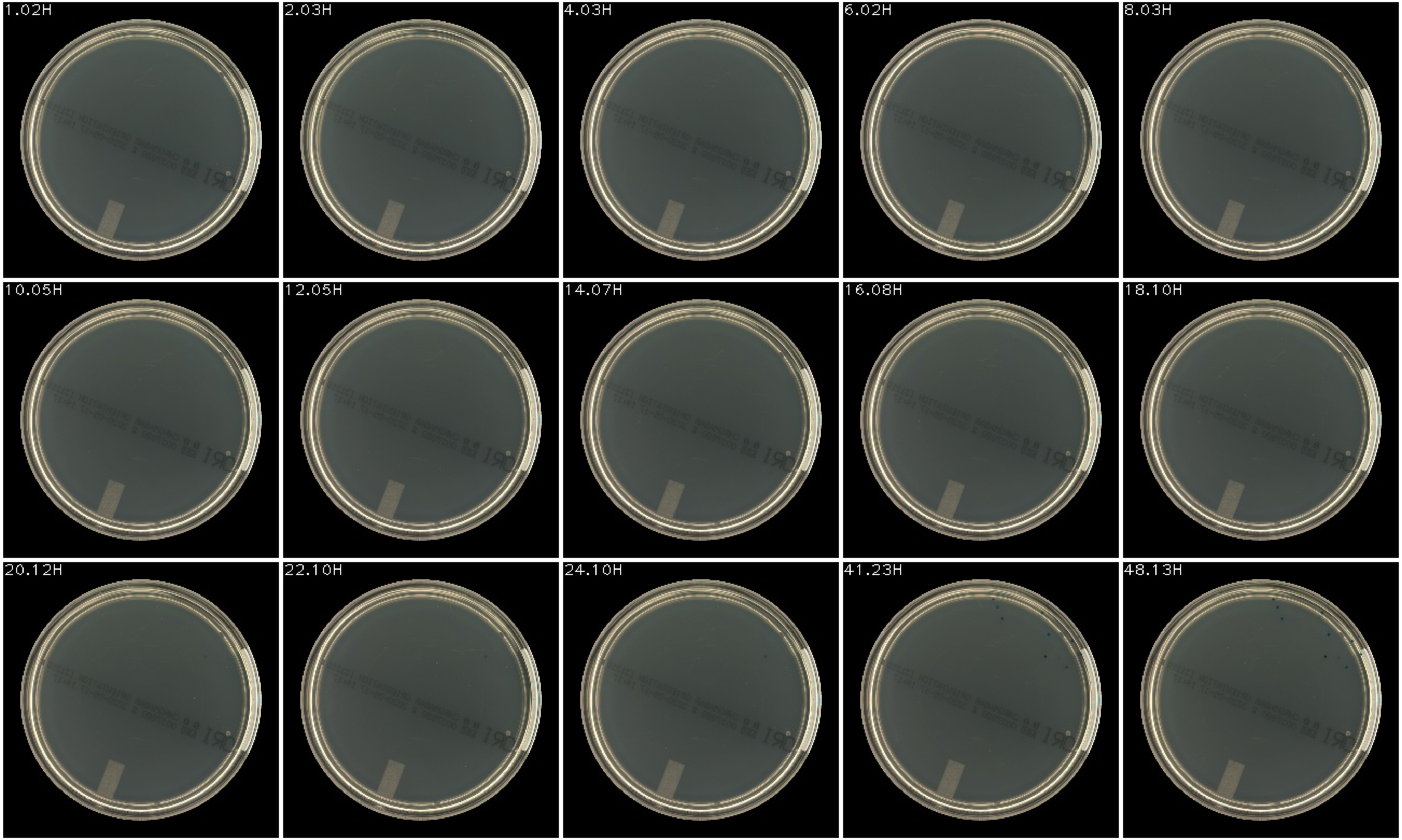

O2-CHROM_ORI-2003-vnuycY5q7qgJpeMLSnk-8/8

TOP_BLACK

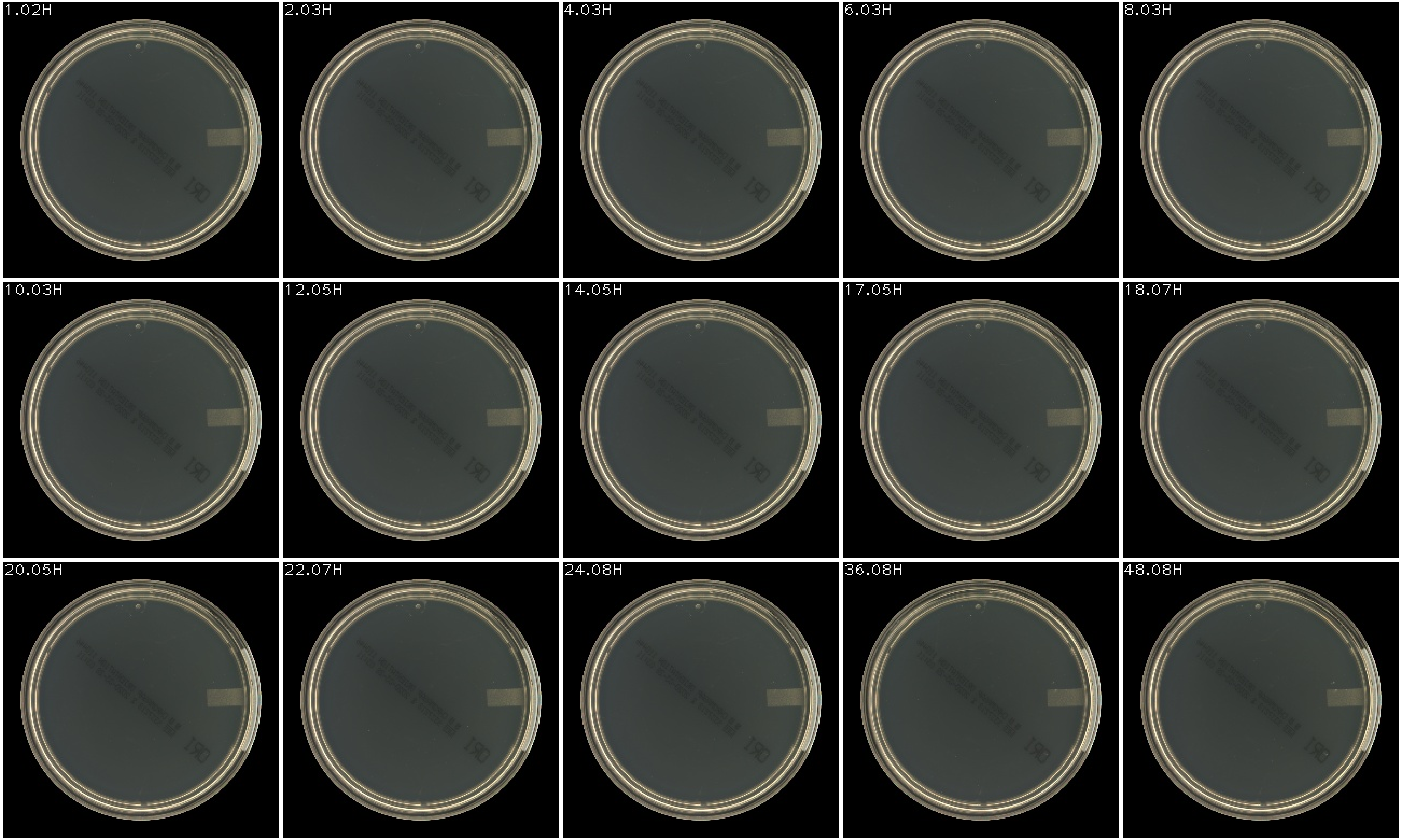

Figure S4

O2-CHROM_ORI-2003-0eEA7CWQPq6A3JhFDrL-1/3

TOP_BLACK

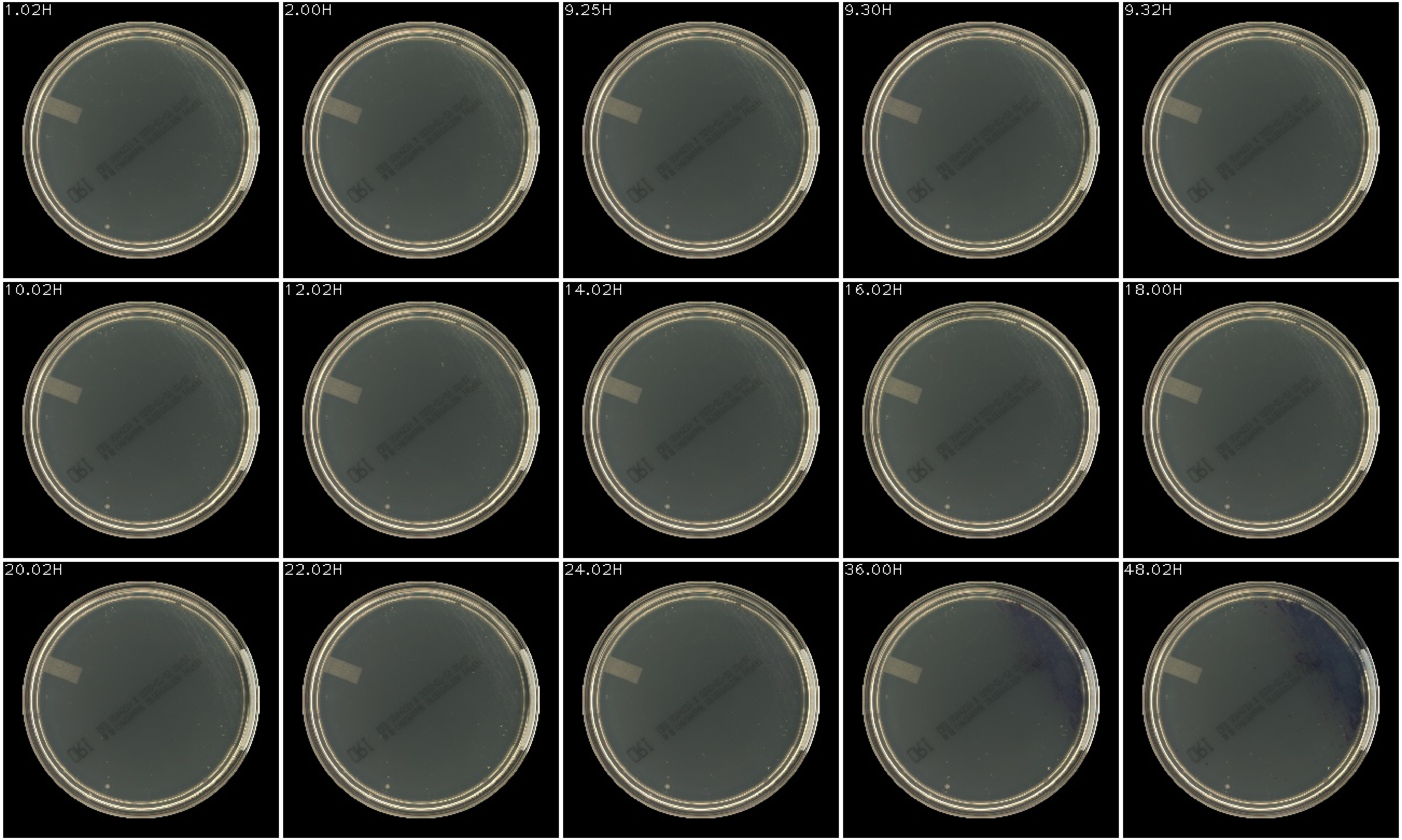

O2-CHROM_ORI-2003-5VVFnrFEY0DMzZNjmyk-2/3

TOP_BLACK

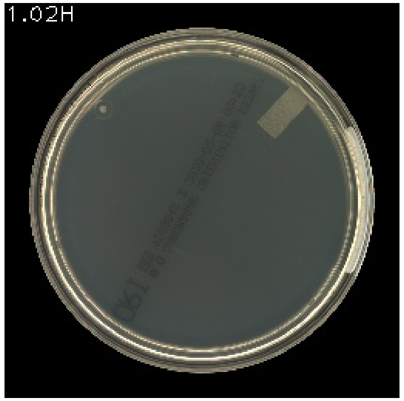

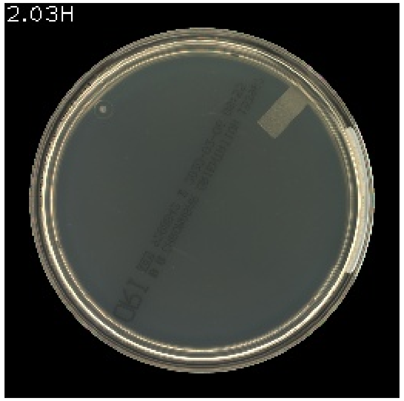

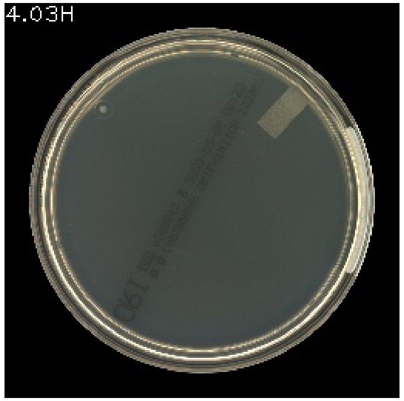

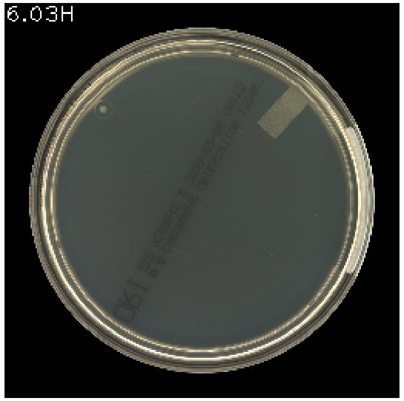

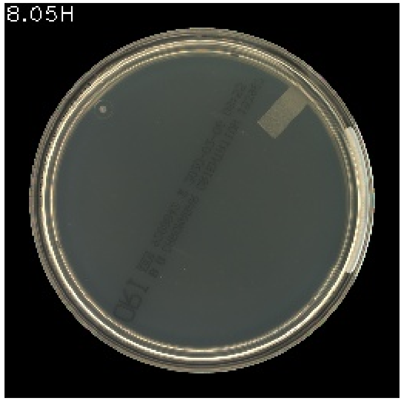

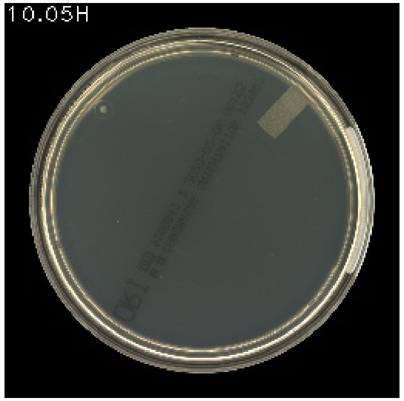

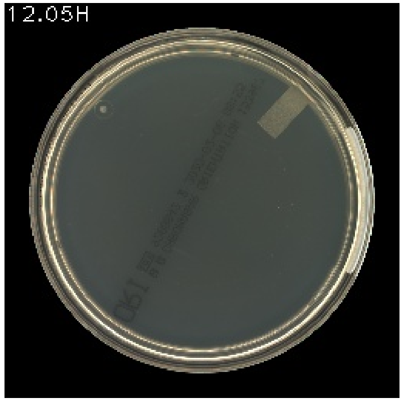

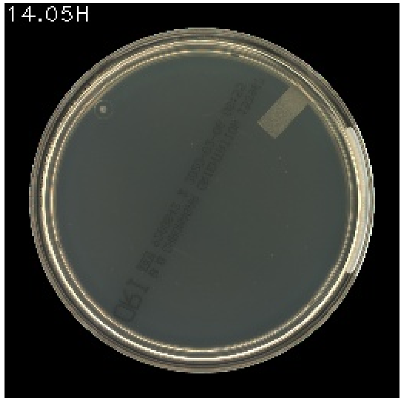

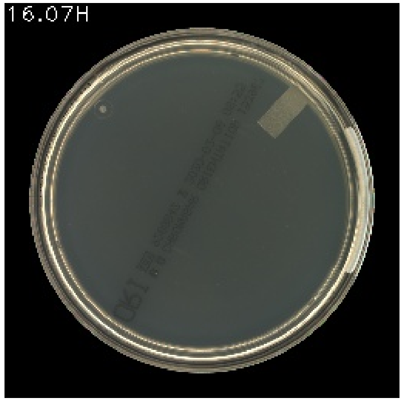

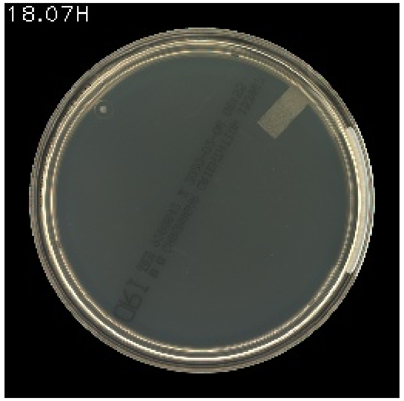

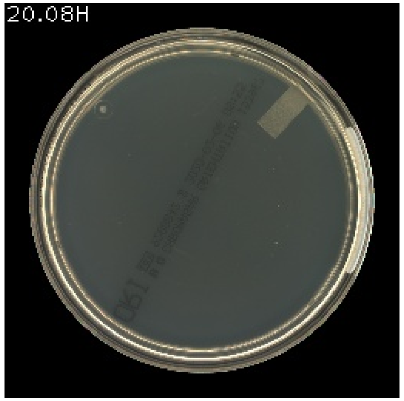

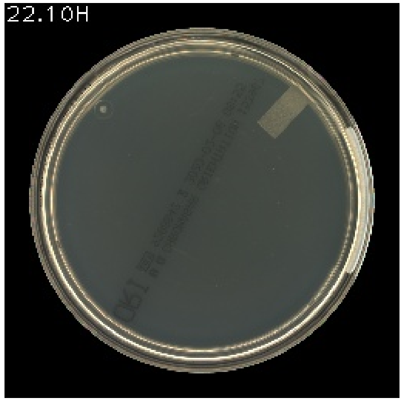

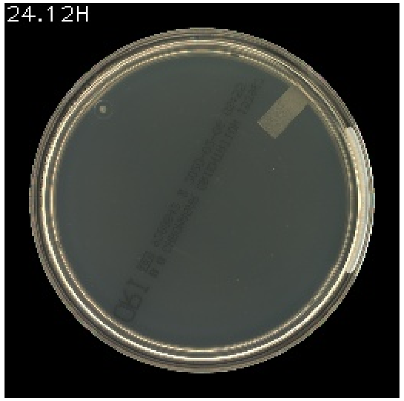

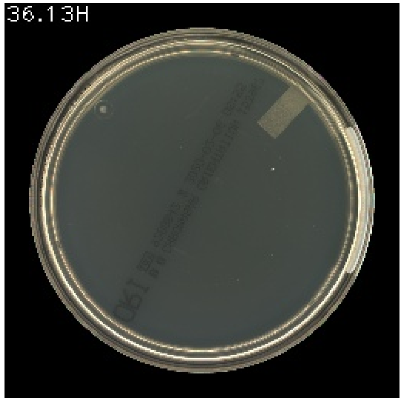

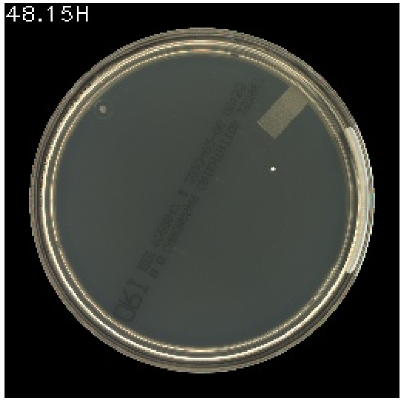

O2-CHROM_ORI-2003-vnuycY5q7qgJpeMLSnk-3/3

TOP_BLACK

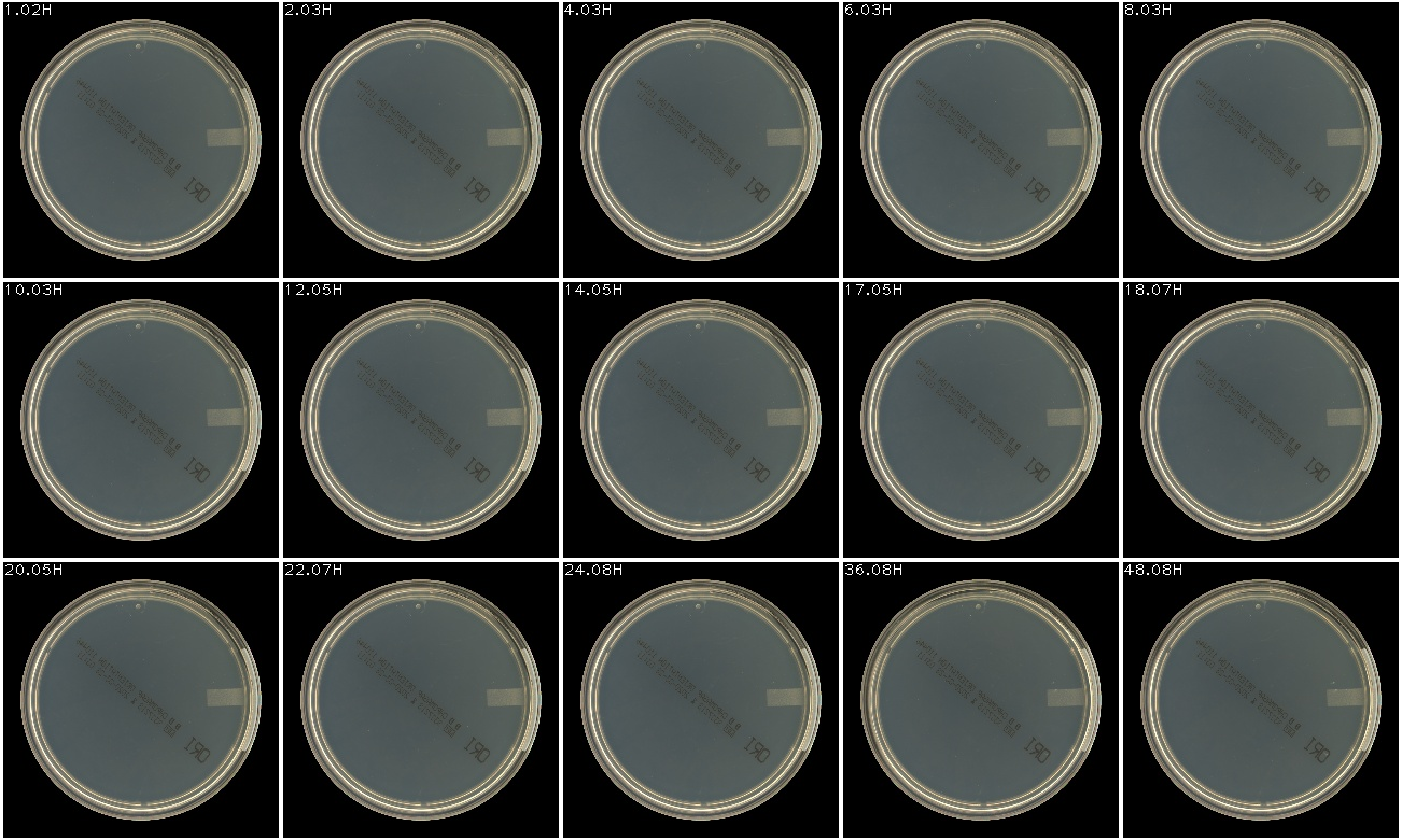

Figure S5

O2-MAC-2003-egfxhmYSsgERxKAKtId-1/1

TOP_BLACK

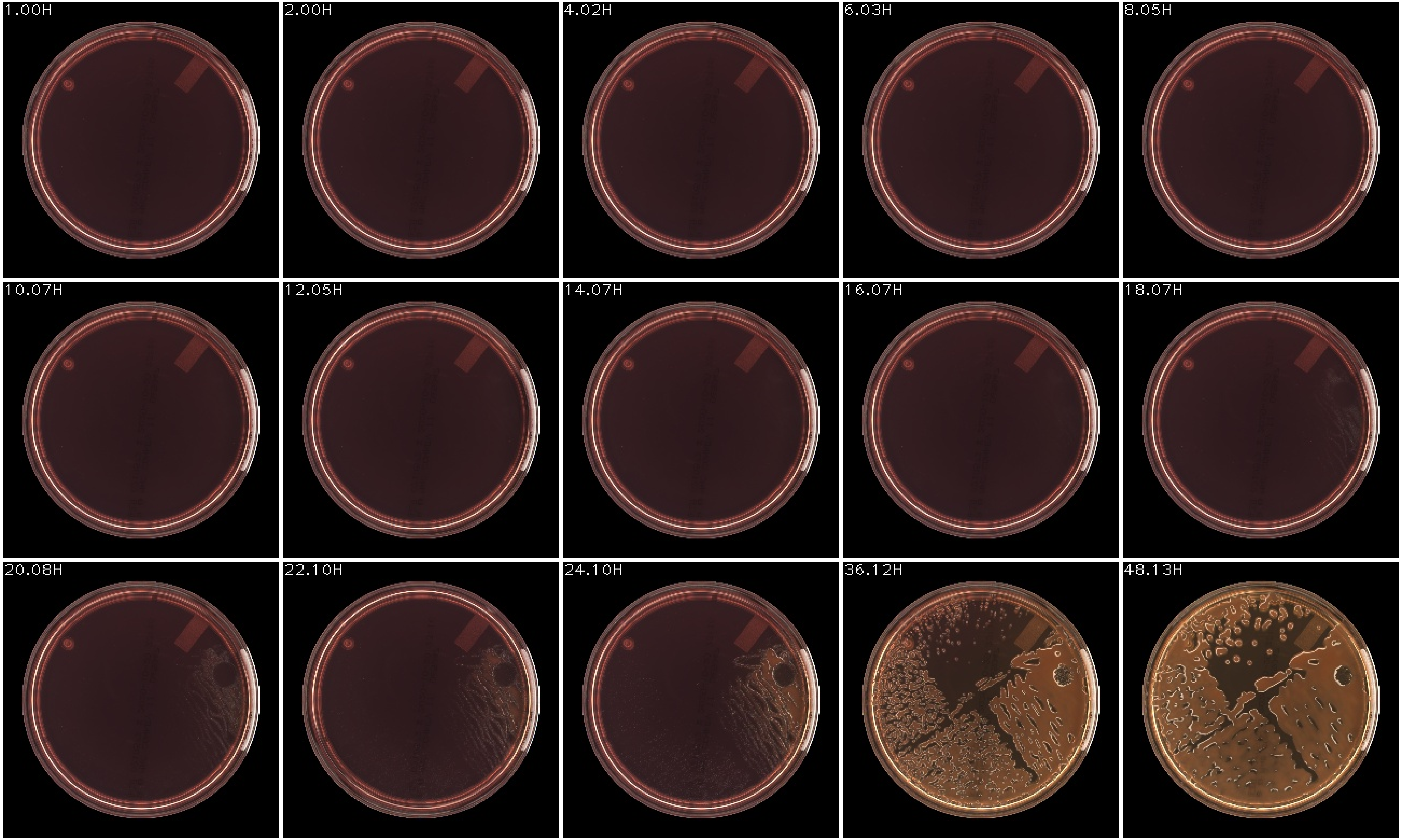

Figure S6

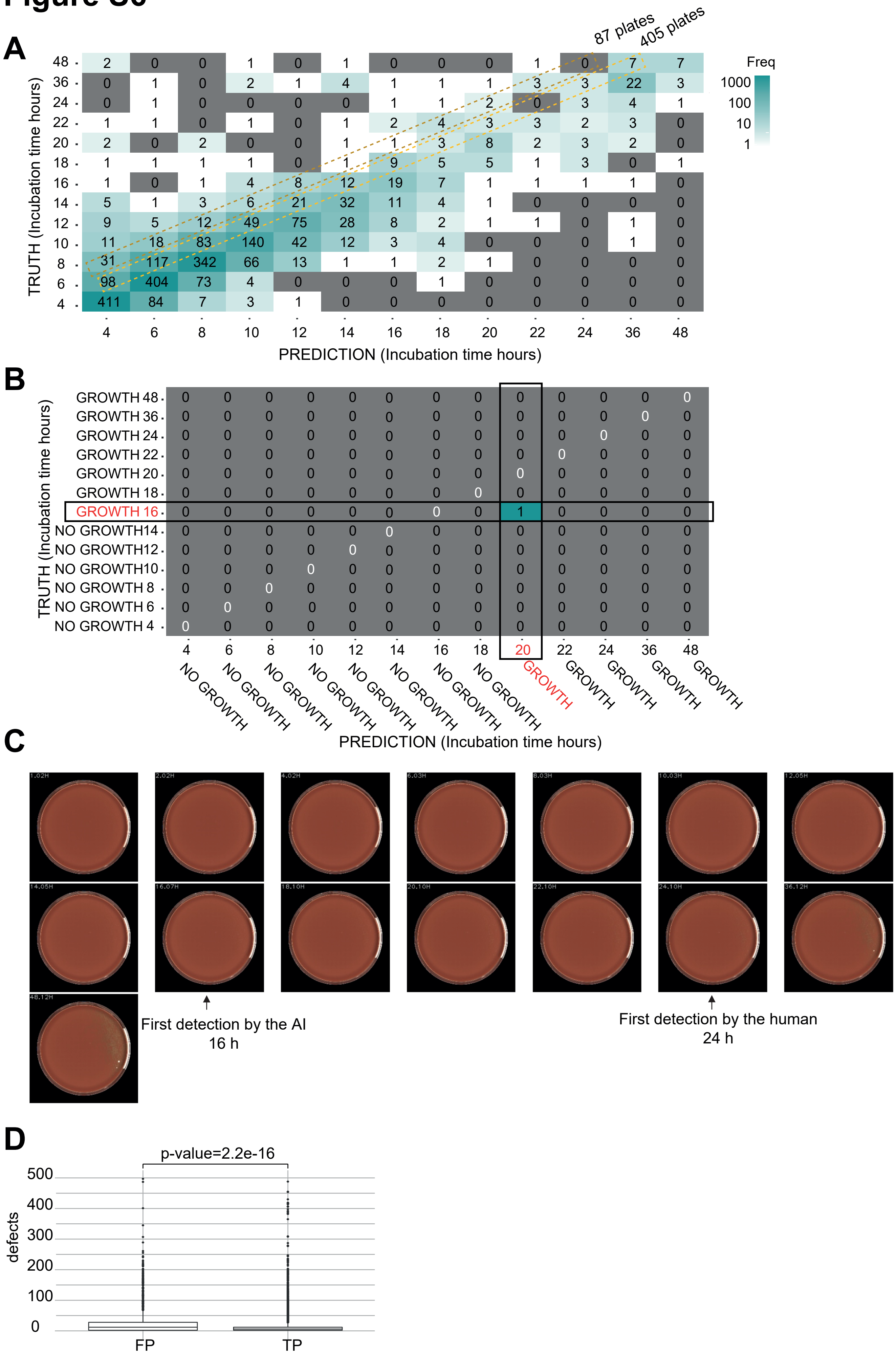
A
87 plates
405 plates
Freq
1000
100
10
1
TRUTH (Incubation time hours)
PREDICTION (Incubation time hours)
B
GROWTH 48
GROWTH 36
GROWTH 24
GROWTH 22
GROWTH 20
GROWTH 18
GROWTH 16
NO GROWTH14
NO GROWTH12
NO GROWTH10
NO GROWTH 8
NO GROWTH 6
NO GROWTH 4
TRUTH (Incubation time hours)
NO GROWTH
GROWTH
PREDICTION (Incubation time hours)
C
First detection by the AI
16 h
First detection by the human
24 h
D
p-value=2.2e-16
defects
FP
TP

Figure S7

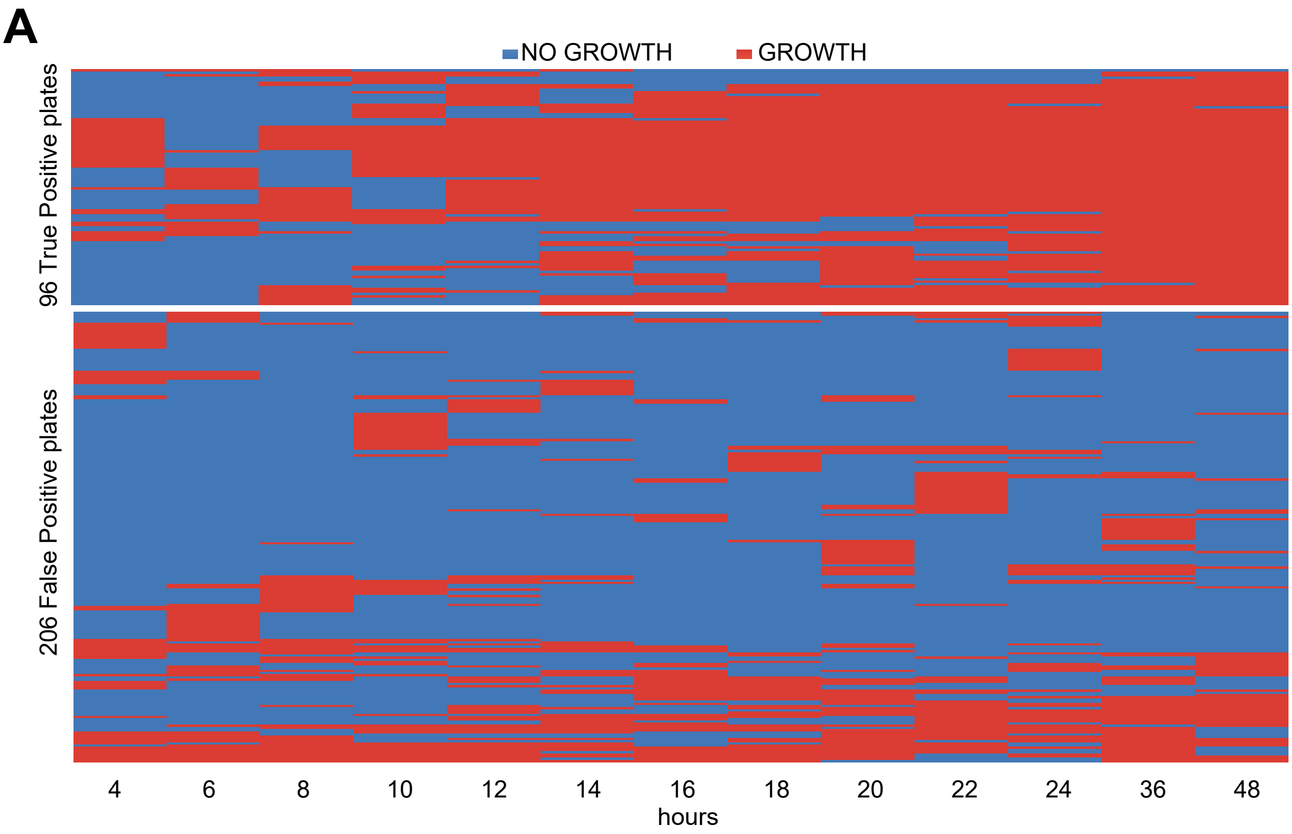
A
NO GROWTH
GROWTH
96 True Positive plates
206 False Positive plates
4
6
8
10
12
14
16
18
20
22
24
36
48
hours

Table S1

		Est	Lower 95% CI	Upper 95% CI		Truth +	Truth -	Total
4 hours	Sensitivity	81.23%	77.55%	84.54%		Truth +	Truth -	Total
	Specificity	93.65%	92.77%	94.45%	Pred +	411	212	623
	Positive predictive value	65.97%	62.10%	69.69%	Pred -	95	3126	3221
	Negative predictive value	97.05%	96.41%	97.61%	Total	506	3338	3844
6 hours	Sensitivity	91.80%	90.01%	93.37%		Truth +	Truth -	Total
	Specificity	91.23%	90.11%	92.26%	Pred +	997	242	1239
	Positive predictive value	80.47%	78.15%	82.64%	Pred -	89	2516	2605
	Negative predictive value	96.58%	95.81%	97.25%	Total	1086	2758	3844
8 hours	Sensitivity	94.22%	92.98%	95.29%		Truth +	Truth -	Total
	Specificity	91.16%	89.89%	92.32%	Pred +	1564	193	1757
	Positive predictive value	89.02%	87.46%	90.44%	Pred -	96	1991	2087
	Negative predictive value	95.40%	94.41%	96.26%	Total	1660	2184	3844
10 hours	Sensitivity	95.49%	94.48%	96.36%		Truth +	Truth -	Total
	Specificity	91.93%	90.60%	93.12%	Pred +	1885	151	2036
	Positive predictive value	92.58%	91.36%	93.68%	Pred -	89	1719	1808
	Negative predictive value	95.08%	93.98%	96.03%	Total	1974	1870	3844
12 hours	Sensitivity	96.40%	95.53%	97.14%		Truth +	Truth -	Total
	Specificity	93.50%	92.21%	94.63%	Pred +	2089	109	2198
	Positive predictive value	95.04%	94.05%	95.91%	Pred -	78	1568	1646
	Negative predictive value	95.26%	94.12%	96.24%	Total	2167	1677	3844
14 hours	Sensitivity	97.51%	96.78%	98.12%		Truth +	Truth -	Total
	Specificity	94.10%	92.82%	95.20%	Pred +	2196	94	2290
	Positive predictive value	95.90%	95.00%	96.67%	Pred -	56	1498	1554
	Negative predictive value	96.40%	95.35%	97.27%	Total	2252	1592	3844
16 hours	Sensitivity	98.09%	97.45%	98.61%		Truth +	Truth -	Total
	Specificity	94.60%	93.35%	95.67%	Pred +	2264	83	2347
	Positive predictive value	96.46%	95.63%	97.17%	Pred -	44	1453	1497
	Negative predictive value	97.06%	96.07%	97.86%	Total	2308	1536	3844
18 hours	Sensitivity	98.76%	98.22%	99.17%		Truth +	Truth -	Total
	Specificity	94.43%	93.15%	95.53%	Pred +	2308	84	2392
	Positive predictive value	96.49%	95.67%	97.19%	Pred -	29	1423	1452
	Negative predictive value	98.00%	97.14%	98.66%	Total	2337	1507	3844
20 hours	Sensitivity	98.86%	98.34%	99.25%		Truth +	Truth -	Total
	Specificity	92.85%	91.42%	94.11%	Pred +	2334	106	2440
	Positive predictive value	95.66%	94.77%	96.43%	Pred -	27	1377	1404
	Negative predictive value	98.08%	97.21%	98.73%	Total	2361	1483	3844
22 hours	Sensitivity	98.87%	98.36%	99.25%		Truth +	Truth -	Total
	Specificity	93.64%	92.26%	94.84%	Pred +	2355	93	2448
	Positive predictive value	96.20%	95.37%	96.92%	Pred -	27	1369	1396
	Negative predictive value	98.07%	97.20%	98.72%	Total	2382	1462	3844
24 hours	Sensitivity	99.08%	98.61%	99.42%		Truth +	Truth -	Total
	Specificity	93.37%	91.97%	94.60%	Pred +	2373	96	2469
	Positive predictive value	96.11%	95.27%	96.84%	Pred -	22	1353	1375
	Negative predictive value	98.40%	97.59%	98.99%	Total	2395	1449	3844
36 hours	Sensitivity	99.59%	99.25%	99.80%		Truth +	Truth -	Total
	Specificity	92.39%	90.88%	93.72%	Pred +	2428	107	2535
	Positive predictive value	95.78%	94.92%	96.53%	Pred -	10	1299	1309
	Negative predictive value	99.24%	98.60%	99.63%	Total	2438	1406	3844
48 hours	Sensitivity	99.59%	99.25%	99.81%		Truth +	Truth -	Total
	Specificity	91.97%	90.41%	93.35%	Pred +	2451	111	2562
	Positive predictive value	95.67%	94.81%	96.42%	Pred -	10	1272	1282
	Negative predictive value	99.14%	98.47%	99.57%	Total	2462	1382	3844

Table S2

		Est	Lower 95% CI	Upper 95% CI		Truth +	Truth -	Total
**CHOC**	**Sensitivity**	96.73%	96.24%	97.17%		**Truth +**	**Truth -**	**Total**
	**Specificity**	84.21%	83.06%	85.31%	**Pred +**	5707	653	6360
	**Positive predictive value**	89.73%	88.96%	90.47%	**Pred -**	193	3483	3676
	**Negative predictive value**	94.75%	93.98%	95.45%	**Total**	5900	4136	10036
**CNA**	**Sensitivity**	97.62%	97.18%	98.01%		**Truth +**	**Truth -**	**Total**
	**Specificity**	93.90%	93.16%	94.58%	**Pred +**	5297	275	5572
	**Positive predictive value**	95.06%	94.46%	95.62%	**Pred -**	129	4231	4360
	**Negative predictive value**	97.04%	96.49%	97.52%	**Total**	5426	4506	9932
**COL**	**Sensitivity**	98.07%	97.71%	98.39%		**Truth +**	**Truth -**	**Total**
	**Specificity**	92.75%	91.83%	93.59%	**Pred +**	6411	251	6662
	**Positive predictive value**	96.23%	95.75%	96.68%	**Pred -**	126	3209	3335
	**Negative predictive value**	96.22%	95.52%	96.84%	**Total**	6537	3460	9997
**ORI**	**Sensitivity**	97.75%	97.33%	98.11%		**Truth +**	**Truth -**	**Total**
	**Specificity**	95.35%	94.67%	95.97%	**Pred +**	5635	195	5830
	**Positive predictive value**	96.66%	96.16%	97.10%	**Pred -**	130	3998	4128
	**Negative predictive value**	96.85%	96.27%	97.36%	**Total**	5765	4193	9958
**MAC**	**Sensitivity**	96.48%	95.72%	97.14%		**Truth +**	**Truth -**	**Total**
	**Specificity**	95.82%	95.34%	96.27%	**Pred +**	2605	307	2912
	**Positive predictive value**	89.46%	88.28%	90.55%	**Pred -**	95	7042	7137
	**Negative predictive value**	98.67%	98.38%	98.92%	**Total**	2700	7349	10049

Table S3

		Est	Lower 95% CI	Upper 95% CI		Truth +	Truth -	Total
**Tissue and Pus**	**Sensitivity**	97.16%	96.54%	97.69%		**Truth +**	**Truth -**	**Total**
	**Specificity**	94.01%	93.50%	94.49%	**Pred +**	3247	545	3792
	**Positive predictive value**	85.63%	84.47%	86.73%	**Pred -**	95	8554	8649
	**Negative predictive value**	98.90%	98.66%	99.11%	**Total**	3342	9099	12441
**Respiratory**	**Sensitivity**	98.07%	97.70%	98.39%		**Truth +**	**Truth -**	**Total**
	**Specificity**	93.30%	92.52%	94.02%	**Pred +**	6392	295	6687
	**Positive predictive value**	95.59%	95.07%	96.07%	**Pred -**	126	4107	4233
	**Negative predictive value**	97.02%	96.47%	97.51%	**Total**	6518	4402	10920
**Superficial wound**	**Sensitivity**	96.28%	94.95%	97.34%		**Truth +**	**Truth -**	**Total**
	**Specificity**	94.01%	92.25%	95.47%	**Pred +**	1010	54	1064
	**Positive predictive value**	94.92%	93.43%	96.16%	**Pred -**	39	847	886
	**Negative predictive value**	95.60%	94.03%	96.85%	**Total**	1049	901	1950
**ENT**	**Sensitivity**	98.82%	97.85%	99.44%		**Truth +**	**Truth -**	**Total**
	**Specificity**	96.73%	94.47%	98.25%	**Pred +**	841	13	854
	**Positive predictive value**	98.48%	97.41%	99.19%	**Pred -**	10	384	394
	**Negative predictive value**	97.46%	95.38%	98.78%	**Total**	851	397	1248
**Urine**	**Sensitivity**	96.61%	96.17%	97.01%		**Truth +**	**Truth -**	**Total**
	**Specificity**	92.63%	91.89%	93.32%	**Pred +**	7124	386	7510
	**Positive predictive value**	94.86%	94.34%	95.35%	**Pred -**	250	4850	5100
	**Negative predictive value**	95.10%	94.47%	95.67%	**Total**	7374	5236	12610
**Urogenital**	**Sensitivity**	97.10%	96.48%	97.65%		**Truth +**	**Truth -**	**Total**
	**Specificity**	83.75%	82.05%	85.34%	**Pred +**	3252	322	3574
	**Positive predictive value**	90.99%	90.00%	91.91%	**Pred -**	97	1659	1756
	**Negative predictive value**	94.48%	93.30%	95.50%	**Total**	3349	1981	5330
**Blood culture**	**Sensitivity**	98.54%	98.11%	98.90%		**Truth +**	**Truth -**	**Total**
	**Specificity**	95.95%	94.87%	96.85%	**Pred +**	3789	66	3855
	**Positive predictive value**	98.29%	97.83%	98.67%	**Pred -**	56	1562	1618
	**Negative predictive value**	96.54%	95.53%	97.38%	**Total**	3845	1628	5473

Table S4

PLATE MEDIA	SPECIMEN GROUP	PRED 48h	TRUTH 48h	COLONIES TYPE	REVISED TRUTH	FALSE NEGATIVE	PRED/TRUTH	4 h	6 h	8 h	10 h	12 h	14 h	16 h	18 h	20 h	22 h	24 h	36 h	48 h
CHOC	2-Urogenital	NO_GROWTH	GROWTH	faint monolayer	GROWTH	YES	PRED	NO_GROWTH	NO_GROWTH	NO_GROWTH	NO_GROWTH	NO_GROWTH	NO_GROWTH	NO_GROWTH	NO_GROWTH	NO_GROWTH	NO_GROWTH	NO_GROWTH	NO_GROWTH	NO_GROWTH
							TRUTH	NO_GROWTH	NO_GROWTH	NO_GROWTH	NO_GROWTH	NO_GROWTH	GROWTH	GROWTH	GROWTH	GROWTH	GROWTH	GROWTH	GROWTH	GROWTH
CHOC	3-Urogenital	NO_GROWTH	GROWTH	one colony	GROWTH	YES	PRED	GROWTH	GROWTH	GROWTH	NO_GROWTH	NO_GROWTH	GROWTH	NO_GROWTH	NO_GROWTH	NO_GROWTH	NO_GROWTH	NO_GROWTH	NO_GROWTH	NO_GROWTH
							TRUTH	NO_GROWTH	NO_GROWTH	NO_GROWTH	NO_GROWTH	NO_GROWTH	NO_GROWTH	NO_GROWTH	NO_GROWTH	NO_GROWTH	NO_GROWTH	NO_GROWTH	NO_GROWTH	GROWTH
CNA	5-Tissue and Pus	NO_GROWTH	GROWTH	one colony	GROWTH	YES	PRED	NO_GROWTH	NO_GROWTH	NO_GROWTH	NO_GROWTH	NO_GROWTH	NO_GROWTH	NO_GROWTH	NO_GROWTH	NO_GROWTH	NO_GROWTH	NO_GROWTH	NO_GROWTH	NO_GROWTH
							TRUTH	NO_GROWTH	NO_GROWTH	NO_GROWTH	NO_GROWTH	GROWTH	GROWTH	GROWTH	GROWTH	GROWTH	GROWTH	GROWTH	GROWTH	GROWTH
CHROM_ORI	1-Urine	NO_GROWTH	GROWTH	two colonies	GROWTH	YES	PRED	NO_GROWTH	NO_GROWTH	NO_GROWTH	NO_GROWTH	NO_GROWTH	NO_GROWTH	NO_GROWTH	NO_GROWTH	NO_GROWTH	NO_GROWTH	NO_GROWTH	NO_GROWTH	NO_GROWTH
							TRUTH	NO_GROWTH	NO_GROWTH	NO_GROWTH	NO_GROWTH	GROWTH	GROWTH	GROWTH	GROWTH	GROWTH	GROWTH	GROWTH	GROWTH	GROWTH
MAC	4-Respiratory	NO_GROWTH	GROWTH	one colony	GROWTH	YES	PRED	NO_GROWTH	NO_GROWTH	NO_GROWTH	NO_GROWTH	NO_GROWTH	NO_GROWTH	NO_GROWTH	NO_GROWTH	NO_GROWTH	NO_GROWTH	NO_GROWTH	NO_GROWTH	NO_GROWTH
							TRUTH	NO_GROWTH	NO_GROWTH	NO_GROWTH	NO_GROWTH	NO_GROWTH	NO_GROWTH	NO_GROWTH	NO_GROWTH	NO_GROWTH	NO_GROWTH	NO_GROWTH	NO_GROWTH	GROWTH
CHOC	Urine	NO_GROWTH	GROWTH	no bacteria	NO_GROWTH	NO	PRED	NO_GROWTH	NO_GROWTH	NO_GROWTH	NO_GROWTH	NO_GROWTH	NO_GROWTH	NO_GROWTH	NO_GROWTH	NO_GROWTH	NO_GROWTH	NO_GROWTH	NO_GROWTH	NO_GROWTH
							TRUTH	NO_GROWTH	NO_GROWTH	NO_GROWTH	NO_GROWTH	NO_GROWTH	NO_GROWTH	NO_GROWTH	NO_GROWTH	NO_GROWTH	NO_GROWTH	NO_GROWTH	GROWTH	GROWTH
CNA	Blood culture	NO_GROWTH	GROWTH	no bacteria	NO_GROWTH	NO	PRED	NO_GROWTH	NO_GROWTH	NO_GROWTH	NO_GROWTH	NO_GROWTH	NO_GROWTH	NO_GROWTH	NO_GROWTH	NO_GROWTH	NO_GROWTH	NO_GROWTH	NO_GROWTH	NO_GROWTH
							TRUTH	NO_GROWTH	NO_GROWTH	NO_GROWTH	NO_GROWTH	NO_GROWTH	NO_GROWTH	NO_GROWTH	NO_GROWTH	NO_GROWTH	NO_GROWTH	NO_GROWTH	NO_GROWTH	GROWTH
COL5BP	Tissue and Pus	NO_GROWTH	GROWTH	no bacteria	NO_GROWTH	NO	PRED	NO_GROWTH	NO_GROWTH	NO_GROWTH	NO_GROWTH	NO_GROWTH	NO_GROWTH	NO_GROWTH	NO_GROWTH	NO_GROWTH	NO_GROWTH	NO_GROWTH	NO_GROWTH	NO_GROWTH
							TRUTH	NO_GROWTH	NO_GROWTH	NO_GROWTH	NO_GROWTH	NO_GROWTH	NO_GROWTH	NO_GROWTH	NO_GROWTH	NO_GROWTH	NO_GROWTH	NO_GROWTH	NO_GROWTH	GROWTH
CHROM_ORI	Urine	NO_GROWTH	GROWTH	no bacteria	NO_GROWTH	NO	PRED	NO_GROWTH	NO_GROWTH	NO_GROWTH	NO_GROWTH	NO_GROWTH	NO_GROWTH	NO_GROWTH	NO_GROWTH	NO_GROWTH	NO_GROWTH	NO_GROWTH	NO_GROWTH	NO_GROWTH
							TRUTH	NO_GROWTH	NO_GROWTH	NO_GROWTH	NO_GROWTH	NO_GROWTH	NO_GROWTH	NO_GROWTH	NO_GROWTH	NO_GROWTH	NO_GROWTH	NO_GROWTH	NO_GROWTH	GROWTH
CHROM_ORI	Urine	NO_GROWTH	GROWTH	no bacteria	NO_GROWTH	NO	PRED	NO_GROWTH	NO_GROWTH	NO_GROWTH	NO_GROWTH	NO_GROWTH	NO_GROWTH	NO_GROWTH	NO_GROWTH	NO_GROWTH	NO_GROWTH	NO_GROWTH	NO_GROWTH	NO_GROWTH
							TRUTH	NO_GROWTH	NO_GROWTH	NO_GROWTH	NO_GROWTH	NO_GROWTH	NO_GROWTH	NO_GROWTH	NO_GROWTH	NO_GROWTH	NO_GROWTH	NO_GROWTH	NO_GROWTH	GROWTH

Table S5

Non-monotonic events	n	percent
1	210	69.77%
2	61	20.27%
3	24	7.97%
4	5	1.66%
5	1	0.33%

Table S6

Plate (Figure 3D)	PRED/TRUTH	4 h	6 h	8 h	10 h	12 h	14 h	16 h	18 h	20 h	22 h	24 h	36 h	48 h
a	PRED	NO_GROWTH	NO_GROWTH	NO_GROWTH	GROWTH	NO_GROWTH	GROWTH	GROWTH	GROWTH	GROWTH	GROWTH	GROWTH	GROWTH	NO_GROWTH
	TRUTH	NO_GROWTH	NO_GROWTH	NO_GROWTH	NO_GROWTH	GROWTH	GROWTH	GROWTH	GROWTH	GROWTH	GROWTH	GROWTH	GROWTH	GROWTH
b	PRED	NO_GROWTH	NO_GROWTH	NO_GROWTH	GROWTH	GROWTH	GROWTH	GROWTH	GROWTH	GROWTH	GROWTH	NO_GROWTH	GROWTH	GROWTH
	TRUTH	NO_GROWTH	NO_GROWTH	NO_GROWTH	GROWTH	GROWTH	GROWTH	GROWTH	GROWTH	GROWTH	GROWTH	GROWTH	GROWTH	GROWTH

Table S7

All plates				
	PREDICTION		TRUTH	
Incubation Time	n	Cumulative %	n	Cumulative %
4	7436	23.23%	6578	20.55%
6	8229	48.94%	7540	44.11%
8	6812	70.23%	7462	67.42%
10	3601	81.48%	4082	80.18%
12	2093	88.02%	2509	88.02%
14	1209	91.80%	1105	91.47%
16	728	94.07%	728	93.74%
18	442	95.45%	377	94.92%
20	299	96.39%	312	95.90%
22	156	96.87%	273	96.75%
24	195	97.48%	169	97.28%
36	533	99.15%	559	99.03%
48	156	100.00%	312	100.00%

Tissue and Pus type				
	PREDICTION		TRUTH	
Incubation Time	n	Cumulative %	n	Cumulative %
4	754	17.90%	637	15.03%
6	1066	43.21%	1105	41.10%
8	1014	67.28%	884	61.96%
10	416	77.16%	546	74.85%
12	273	83.64%	312	82.21%
14	208	88.58%	260	88.34%
16	117	91.36%	65	89.88%
18	52	92.59%	65	91.41%
20	78	94.44%	78	93.25%
22	13	94.75%	39	94.17%
24	65	96.30%	39	95.09%
36	143	99.69%	117	97.85%
48	13	100.00%	91	100.00%

Blood culture specimen				
	PREDICTION		TRUTH	
Incubation Time	n	Cumulative %	n	Cumulative %
4	2535	62.90%	2665	66.13%
6	1014	88.06%	936	89.35%
8	299	95.48%	312	97.10%
10	104	98.06%	39	98.06%
12	13	98.39%	13	98.39%
14	0	98.39%	0	98.39%
16	13	98.71%	0	98.39%
18	0	98.71%	0	98.39%
20	0	98.71%	0	98.39%
22	26	99.35%	26	99.03%
24	0	99.35%	0	99.03%
36	13	100.00%	26	99.68%
48			13	100.00%
